# Chemical Modification of Dental Dimethacrylate Copolymer with Tetramethylxylylene Diisocyanate-Based Quaternary Ammonium Urethane-Dimethacrylates—Physicochemical, Mechanical, and Antibacterial Properties

**DOI:** 10.3390/ma17020298

**Published:** 2024-01-07

**Authors:** Patryk Drejka, Marta Chrószcz-Porębska, Alicja Kazek-Kęsik, Grzegorz Chladek, Izabela Barszczewska-Rybarek

**Affiliations:** 1 Department of Physical Chemistry and Technology of Polymers, Faculty of Chemistry, Silesian University of Technology, Strzody 9 Str., 44-100 Gliwice, Poland; patryk.drejka@polsl.pl (P.D.); marta.chroszcz.porebska@gmail.com (M.C.-P.); 2 Department of Inorganic Chemistry, Analytical Chemistry and Electrochemistry, Faculty of Chemistry, Silesian University of Technology, Krzywoustego 6 Str., 44-100 Gliwice, Poland; alicja.kazek-kesik@polsl.pl; 3 Biotechnology Centre, Silesian University of Technology, Krzywoustego 8 Str., 44-100 Gliwice, Poland; 4 Department of Engineering Materials and Biomaterials, Faculty of Mechanical Engineering, Silesian University of Technology, Konarskiego 18A Str., 44-100 Gliwice, Poland; grzegorz.chladek@polsl.pl

**Keywords:** dental resin, urethane-dimethacrylate, quaternary ammonium, copolymerization, photocuring, antibacterial properties, physicochemical properties, mechanical properties

## Abstract

In this study, two novel quaternary ammonium urethane-dimethacrylates (QAUDMAs) were designed for potential use as comonomers in antibacterial dental composite restorative materials. QAUDMAs were synthesized via the reaction of 1,3-bis(1-isocyanato-1-methylethyl)benzene with 2-(methacryloyloxy)ethyl-2-decylhydroxyethylmethylammonium bromide (QA10+TMXDI) and 2-(methacryloyloxy)ethyl-2-dodecylhydroxyethylmethylammonium bromide (QA12+TMXDI). Their compositions with common dental dimethacrylates comprising QAUDMA 20 wt.%, urethane-dimethacrylate monomer (UDMA) 20 wt.%, bisphenol A glycerolate dimethacrylate (Bis-GMA) 40 wt.%, and triethylene glycol dimethacrylate (TEGDMA) 20 wt.%, were photocured. The achieved copolymers were characterized for their physicochemical and mechanical properties, including their degree of conversion (*DC*), glass transition temperature (*T_g_*), polymerization shrinkage (*S*), water contact angle (*WCA*), flexural modulus (*E*), flexural strength (*FS*), hardness (*HB*), water sorption (*WS*), and water leachability (*WL*). The antibacterial activity of the copolymers was characterized by the minimum bactericidal concentration (*MBC*) and minimum inhibitory concentration (*MIC*) against *Staphylococcus aureus* and *Escherichia coli*. The achieved results were compared to the properties of a typical dental copolymer comprising UDMA 40 wt.%, Bis-GMA 40 wt.%, and TEGDMA 20 wt.%. The introduction of QAUDMAs did not deteriorate physicochemical and mechanical properties. The *WS* and *WL* increased; however, they were still satisfactory. The copolymer comprising QA10+TMXDI showed a higher antibacterial effect than that comprising QA12+TMXDI and that of the reference copolymer.

## 1. Introduction

Faced with the increase in the dental caries scale [1,2], it is important to find effective solutions for curing this disease. Therefore, in recent years, an upward trend in the development of antimicrobial dental composite restorative materials (DCRMs) is observed [3,4,5,6,7,8,9,10,11,12,13,14,15]. The elementary concept of providing DCRMs with antimicrobial properties assumes physically dispersing bioactive substances (such as antibiotics, antimicrobial enzymes, chlorohexidine, triclosan, metals, metal oxides, and the nanoparticles of quaternized polyethyleneimine) in available dental materials [5,6,7,8,9,10,11,12,14]. As these types of biocides can easily elute from a material, the material achieved shows only a short-term antimicrobial effect. It quickly loses its mechanical and antimicrobial properties and causes cytotoxic effects [16,17].

Another concept of providing DCRMs with antimicrobial properties is based on the covalent incorporation of a monomeric biocide into the polymer network structure, constituting the DCRM matrix. It involves the copolymerization of universal dental dimethacrylates (DMAs), such as bisphenol A glycerolate dimethacrylate (Bis-GMA), its ethoxylated derivative (Bis-EMA), urethane-dimethacrylate monomer (UDMA), and triethylene glycol dimethacrylate (TEGDMA), with dimethacrylates having bioactive groups, of which dimethacrylates with quaternary ammonium (QA) groups (QADMAs) are the best known [18,19,20,21,22]. The QADMA content in a copolymer ranges from a few to several dozen percent. This alternative offers the stable, non-leaching, and long-term antimicrobial action of a DCRM [23,24]. The huge advantages of monomeric antimicrobials include lower cytotoxic effects in comparison to physically dispersed antibacterial agents [16,25] and an alternative to antibiotics, whose application should be limited due to the increasing resistance of microorganisms [26,27,28]. The aforementioned features of QADMAs explain the great interest among scientists in designing novel monomer structures containing QA groups and the development of related materials.

The following series of QADMAs are described in the literature:

(i) dimethacrylate derivatives of *N*-methyldiethanolamine (MDEA) with one central quaternary ammonium group (QAn+MDEA, where n is the number of carbon atoms in the *N*-alkyl substituent of the QA group). QA12+MDEA and QA16+MDEA were studied [29,30];
(ii) dimethacrylate derivatives of *N*,*N*-dimethylaminoethyl methacrylate (DMAEMA) with two QA groups separated with a central aliphatic chain (QAn+DMAEMA, where n is the number of carbon atoms in the chain separating QA groups). QA groups in these monomers were substituted with two methyl groups. QA4+DMAEMA and QA6+DMAEMA were studied [31];
(iii) the Bis-GMA quaternary ammonium derivative with two QA groups (QAn+bis-GMA, where n is the number of carbon atoms in the *N*-alkyl substituent of the QA group). QA6+bis-GMA was studied [32];
(iv) fully aliphatic urethane-dimethacrylates with one central QA group, based on *N*-methyldiethanolamine (MDEA) and 2-isocyanatoethyl methacrylate (IEM) (QAn+MDEA+IEM, where n is the number of carbon atoms in the *N*-alkyl substituents of the QA group). QAn+MDEA+IEMs with n of 12, 14, 16, and 18 were studied [33,34];
(v) urethane-dimethacrylates with one central QA group, based on N-methyldiethanolamine (MDEA) and cycloaliphatic isophorone diisocyanate (IPDI) (QAn+MDEA+IPDI, where n is the number of carbon atoms in the *N*-alkyl substituent of the QA group). QAn+MDEA+IPDIs with n of 12, 14, 16, and 18 were studied [35,36];
(vi) quaternary ammonium analogues of the urethane-dimethacrylate monomer with two QA groups (QAn+TMDI, where n is the number of carbon atoms in the *N*-alkyl substituent of the QA group). QAn+TMDI with n of 8, 10, 12, 14, 16, and 18 were studied [37,38,39,40,41,42].

QAn+MDEAs showed high antibacterial activity against many bacteria strains, including *S. aureus*, *Streptococcus mutans*, *Actinomyces viscosus*, *Lactobacillus acidophilus*, *Streptococcus sanguinis*, *Porphyromonas gingivalis*, *Prevotella melaninogenica*, and *Enterococcus faecalis.* In addition, these monomers showed cytotoxicity lower than Bis-GMA [29]. The copolymer of QAn+MDEA 10 wt.% with Bis-GMA/TEGDMA 90 wt.% showed antibacterial activity against *S. mutans* [30]. QAn+DMAEMA 1 wt.% was used to modify a commercial adhesive resin (Tetric N-Bond) and the resulting material also showed antibacterial activity against *S. mutans* [31]. The introduction of QAn+Bis-GMA 5 wt.% into the Bis-GMA/TEGDMA formulation resulted in a copolymer with antibacterial activity against *E. coli*, *S. aureus*, *S. mutans*, and *Bacillus subtillis*. The antifungal activity of the modified copolymer against *Candida albicans* was observed too. As the QAn+Bis-GMA concentration increased, the antimicrobial activity increased; however, it was accompanied by an increase in cytotoxicity and the deterioration of mechanical properties [32]. The modification of the same Bis-GMA/TEGDMA formulation with QAn+MDEA+IEM 5 wt.% resulted in an antibacterial copolymer only for QA16+MDEA+IEM. It showed antibacterial activity against *S. mutans*. All other copolymers gained antibacterial activity as the QAn+MDEA+IEMs concentration increased to 10 wt.%, but this was accompanied by the deterioration in mechanical properties [33,34]. The QAn+MDEA+IPDI 50 wt.% and TEGDMA 50 wt.% copolymers also showed high antibacterial activity against *S. mutans*. However, the disadvantages of these copolymers included a low flexural strength, low modulus of elasticity, and high water sorption [35,36]. By reducing the QAn+MDEA+IPDI content to 30 wt.% and using a formulation of Bis-GMA/TEGDMA 70 wt.%, an improvement in mechanical properties was achieved [36]. QAn+TMDIs and their copolymers were the most extensively studied [37,38,39,40,41,42]. Copolymers of QAn+TMDI 60 wt.% and TEGDMA 40 wt.% were characterized by very high antibacterial activity against *S. aureus* and *E. coli*. However, they had insufficiently good mechanical parameters, excessively high water sorption, and water leachability [38,39]. Copolymers of QAn+TMDI 40 wt.%, Bis-GMA 40 wt.%, and TEGDMA 20 wt.% also showed high antimicrobial activity against *S. aureus* and *E. coli* [40]. In addition, antifungal activity against *C. albicans* was observed for these copolymers [41]. Many of their physicochemical, mechanical, and biological parameters were good. However, the detailed analysis showed that an increase in the *N*-alkyl substituent length resulted in a decrease in the antibacterial activity, and the deterioration of mechanical properties [40,41,42]. This analysis led to the conclusion that copolymers based on QA10+TMDI and QA12+TMDI contain the optimum combination of properties [40].

The promising results obtained for copolymers based on QA10+TMDI and QA12+TMDI motivated us to replace the TMDI core with a 1,3-bis(1-isocyanate-1-methylethyl)benzene (TMXDI) core to obtain new urethane-dimethacrylate monomers with two QA groups located in the wings and 1,3-phenylene ring located in the core. We formulated a hypothesis that the copolymerization of the achieved quaternary ammonium urethane-dimethacrylates (QAUDMAs) with UDMA, Bis-GMA, and TEGDMA results in an antibacterial copolymer with good physicochemical and mechanical properties. Therefore, the goal of this work was to synthesize two novel urethane-dimethacrylates with the TMXDI core and two methacrylate-ended wings comprising the QA group in the middle. The QA groups were substituted with an *N*-alkyl chain of 10 and 12 carbon atoms (QA10+TMXDI and QA12+TMXDI, respectively). The two novel monomers were compounded with common dental dimethacrylates in the following weight fractions: QAUDMA 20 wt.%, UDMA 20 wt.%, Bis-GMA 40 wt.%, and TEGDMA 20 wt.%. The two novel monomers were also photocured. The novel monomers, as well as their compositions with dental dimethacrylates, were characterized for molecular weight (*MW*), concentration of double bonds (*x_DB_*), viscosity (*η*), refractive index (*RI*), and density (*d_m_*). The degree of conversion (*DC*), density (*d_p_*), polymerization shrinkage (*S*), glass transition temperature (*T_g_*), water contact angle (*WCA*), water sorption (*WS*), water solubility (*SL*), hardness (*HB*), flexural strength (*FS*), flexural modulus (*E*), minimum bactericidal concentration (*MBC*), and minimum inhibitory concentration (*MIC*) were all measured for the resulting copolymers (the first comprising QA10+TMXDI (20(QA10+TMXDI)_p_) and the second comprising QA12+TMXDI (20(QA12+TMXDI)_p_). The results were subjected to a comparative analysis of the properties of the standard dental copolymer consisting of UDMA 40 wt.%, Bis-GMA 40 wt.%, and TEGDMA 20 wt.% (40(UDMA)_p_).

The novelty of this work combines the development of two new urethane-dimethacrylate monomers with quaternary ammonium groups (QAUDMAs) and the complex physicochemical, mechanical, and antibacterial characteristics of their copolymers with common dimethacrylates used in dentistry. So far, QAUDMAs synthesized from 1,3-bis(1-isocyanato-1-methylethyl)benzene have not been described in the literature.

The development of such materials is very important for dental science and may contribute to reducing the scale of dental carries in the future.

## 2. Materials and Methods

### 2.1. Materials

Bisphenol A-glycidyl dimethacrylate (Bis-GMA), triethylene glycol dimethacrylate (TEGDMA), urethane-dimethacrylate (UDMA), 1,3-bis(1-isocyanato-1-methylethyl)benzene (TMXDI), phenothiazine (PTZ), camphorquinone (CQ), *N*,*N*-dimethylaminoethyl methacrylate (DMAEMA), potassium bromide (KBr, FT-IR grade), and tetramethylsilane (TMS) were purchased from Sigma-Aldrich, St. Louis, MO, USA. Methyl methacrylate (MMA), *N*-methyldiethanolamine (MDEA), decyl bromide (DB), and dodecyl bromide (DDB) were purchased from Acros Organics, Geel, Belgium. Dibutyltin dilaurate (DBTDL) was purchased from Fluka, Charlotte, NC, USA. Deuterated chloroform (CDCl_3_) and deuterated dichloromethane (CD_2_Cl_2_) were purchased from Deutero GMBH, Kastellaun, Germany. Potassium carbonate (K_2_CO_3_), and magnesium sulfate (MgSO_4_) were purchased from Chempur, Piekary Śląskie, Poland. Toluene, chloroform, and dichloromethane were purchased from Stanlab, Lublin, Poland. All chemicals were used as received.

### 2.2. Chemical Syntheses

#### 2.2.1. *N*,*N*-(2-Hydroxyethyl)methylaminoethyl Methacrylate (HAMA)

The transesterification reaction reagents—MMA 1.00 mol (100.12 g), and MDEA 0.67 mol (79.85 g), the catalyst—K_2_CO_3_ 8 wt.% (14.40 g), the polymerization inhibitor—PTZ 0.05% (0.09 g) and the solvent—toluene 400 cm^3^ were introduced into a single-necked round-bottom flask equipped with a Vigreux column and distillation head. The reaction mixture was brought to a boil. The equilibrium of the reaction was moved in favor of HAMA by the continuous collection of a distillate consisting of an azeotropic mixture of methanol (condensation reaction by-product), MMA (used in excess), and toluene (solvent). The reaction was carried out until the temperature at the column head reached 100 °C, which was achieved after 2.5 h. The cooled reaction mixture was filtered and washed three times with distilled water in a 1:2 volume ratio (HAMA and MDEA were soluble in water). The aqueous fractions were combined and extracted three times with chloroform in a 1:3 volume ratio (only HAMA was soluble in chloroform; as MDEA was water-insoluble, it remained in a water fraction). The chloroform fractions, containing only HAMA, were also combined, dried with MgSO_4_ overnight, and chloroform was evaporated on a rotary evaporator under reduced pressure (30 and then 3 mbar). The obtained raw product was subjected to vacuum distillation (3 mbar), collecting a boiling fraction from 110 to 130 °C. The process yielded HAMA 14%.

#### 2.2.2. 2-(Methacryloyloxy)ethyl-2-hydroxyethylmethylalkylammonium Bromides (QAHAMA-n, Where n Is the Number of Carbon Atoms in the *N*-Alkyl Substituent)

The Menshutkin reaction reagents—HAMA 0.107 mol (20.00 g), and alkyl bromide 0.107 mol (DB 23.66 g and DDB 26.67 g), and the polymerization inhibitor—PTZ 0.05 wt.% (0.022 and 0.023 g, respectively, in the reaction with DB and DDB) were introduced into a three-necked round-bottom flask equipped with a mechanical stirrer, reflux condenser, and thermometer. The reaction was carried out at 80 °C for 90 h. The reaction yielded 100% of 2-(methacryloyloxy)ethyl-2-decylhydroxyethylmethylammonium bromide (QAHAMA-10) and 2-(methacryloyloxy)ethyl-2-dodecylhydroxyethylmethylammonium bromide (QAHAMA-12).

#### 2.2.3. Quaternary Ammonium Urethane-Dimethacrylates (QAUDMA)

A 50% solution of an addition reaction reagent—QAHAMA-n 0.070 mol (QAHAMA-10 28.50 g and QAHAMA-12 28.6 g), a catalyst—DBTDL 0.035 wt.% (0.013 g) and a solvent—dichloromethane 21 cm^3^ were introduced into a three-necked round-bottom flask equipped with a reflux condenser, thermometer, and dropping funnel. A 50% solution of the second reagent, composed of TMXDI 0.035 mol (8.55 g) and dichloromethane 6.5 cm^3^, was placed in a dropping funnel. The flask content was brought to a boil (approximately 42 °C), and then a TMXDI solution was added dropwise for 1.5 h. The reaction was carried out for 5 h, maintaining the reaction mixture at the boiling point. Dichloromethane was evaporated on a rotary evaporator under reduced pressure (30 and then 3 mbar). A light-yellow, viscous, liquid substance remained in the flask, constituting the product. The flask with the product was placed in the laboratory dryer (SLW 53 STD, POL-EKO, Wodzisław Śląski, Poland) and heated at 42 °C for 24 h. The reaction yielded 100% both QAUDMAs, i.e., the addition reaction of TMXDI and QAHAMA-10 yielded QA10+TMXDI, and that of QAHAMA-12 yielded QA12+TMXDI.

### 2.3. Curing Procedure and Sample Preparation

Two experimental monomer compositions were prepared using mechanical stirring at 50 °C. They consisted of QAUDMA (QA10+TMXDI and QA12+TMXDI) 20 wt.%, UDMA 20 wt.%, Bis-GMA 40 wt.%, and TEGDMA 20 wt.%. The reference composition consisted of UDMA 40 wt.%, Bis-GMA 40 wt.%, and TEGDMA 20 wt.%. The components of the initiating system, comprising CQ 0.4 wt.% and DMAEMA 1 wt.%, were introduced into homogeneous monomer mixtures and stirring was continued at the same temperature until CQ dissolved. The achieved compositions were poured into the molds, covered with the PET foil, and irradiated with the UV–VIS lamp (Ultra Vitalux 300, Osram, Munich, Germany) at room temperature for 1 h. The lamp emitted radiation in the range of 280 to 780 nm and radiation exitance of 2400 mW/cm^2^. The achieved casts were processed following the guidelines for specimen preparation for testing particular properties. Each specimen was polished with fine sandpaper before experiments.

### 2.4. Nuclear Magnetic Resonance Spectroscopy (NMR)

The NMR 300 MHz spectrometer (UNITY/INOVA, Varian, Palo Alto, CA, USA) was used to collect 256 scan ^1^H spectra and 40,000 scan ^13^C NMR spectra of products achieved. CD_2_Cl_2_ and CDCl_3_ were used as solvents and TMS was used as an internal standard.

### 2.5. Fourier Transform Infrared Spectroscopy (FTIR)

The FTIR spectrometer (Spectrum Two, Perkin-Elmer, Waltham, MA, USA) was used to collect spectra (128 scans, 1 cm^−1^ resolution).

Monomers were tested as a very thin layer between two KBr pellets. Copolymers were powdered, sieved to a diameter of less than 25 µm, and dispersed in KBr pellets.

The degree of conversion (*DC*) in copolymers was calculated from the following equation:

(1) $$DC=1-\frac{{\left(\frac{A_{C=C}}{A_{Ar}}\right)}_{polymer}}{{\left(\frac{A_{C=C}}{A_{Ar}}\right)}_{monomer}}$$

The variables in the equation are defined as follows:

*A_C=C_*—the absorbance of the stretching vibration band of the carbon–carbon double bond in the methacrylate group at 1636 cm^−1^;
*A_Ar_*—the absorbance of the aromatic stretching skeletal vibrations band at 1608 cm^−1^.

### 2.6. Viscosity

The rotating viscometer (Visco Star Plus L, Brookfield Fungilab Viscometer, Barcelona, Spain) was used to determine the viscosity (η). The procedure was carried out at 25 °C according to the ISO 2555-2018 standard [43].

### 2.7. Refractive Index

The digital refractometer (DR 6100T, Krüss Optronic, Hamburg, Germany) was used to determine the refractive index (*RI*). The procedure was carried out according to the ISO 489:2022 standard [44].

### 2.8. Density and Polymerization Shrinkage

The density of monomers (*d_m_*) was determined with the 1 mL pycnometer. The density of copolymers (*d_p_*) was determined with the analytical balance (XP Balance, Mettler Toledo, Greifensee, Switzerland) analytical balance equipped with the density determination kit.

The polymerization shrinkage (*S*) was calculated from the following equation:

(2) $$S\left(\%\right)=\left(1-\frac{d_{m}}{d_{p}}\right)\times 100$$

The variables in the equation are defined as follows:

*d_m_*—the monomer density;
*d_p_*—the polymer density.

### 2.9. Differential Scanning Calorimetry (DSC)

The differential scanning calorimeter (DSC 3, Mettler Toledo, Greifensee, Switzerland) was used in the measurements, utilizing powdered polymer samples and standard aluminum crucibles. The 10 K/min heating rate, 0 to 200 °C temperature range, and air atmosphere were used in all experiments.

The glass transition temperature (*T_g_*) of copolymers was read off the DSC curve as the midpoint of the transition region, following the 11357-2:2020 standard [45].

### 2.10. Water Contact Angle

The goniometer (OCA 15EC, Data Physics, Filderstadt, Germany) was used to determine the water contact angle (*WCA*) of copolymer surfaces. The analysis was performed on disc-like specimens with dimensions of 15 mm × 1.5 mm (diameter × thickness) utilizing the sessile drop method and 4 µL of deionized water. The measurement was performed immediately after the drop was placed on the sample surface.

### 2.11. Water Sorption and Solubility

The water sorption (*WS*) and solubility (*SL*) of polymers were determined according to the guidelines of the ISO 4049:2019 standard [46] on disc-like specimens with dimensions of 15 mm × 1.5 mm (diameter × thickness). The analytical balance (XP Balance, Mettler Toledo, Greifensee, Switzerland) was used in experiments.

The specimens were dried to constant weight (*m*_0_) in a laboratory dryer (SLW 53 STD, POL-EKO, Wodzisław Śląski, Poland) and immersed in deionized water at room temperature for 7 days. After that, samples were removed from the water, dried with blotting paper, weighed (*m*_1_), and dried again to a constant weight (*m*_2_).

*WS* and *SL* were calculated using the following equations:

(3) WS μgmm3=m1−m0V,

(4) SL μgmm3=m0−m2V

The variables in the equation are defined as follows:

m_0_—the initial mass of the dried samples;
*m*_1_—the mass of the swollen samples;
*m*_2_—the mass of the dried samples after immersion in water;
*V*—the initial volume of the dried samples.

### 2.12. Mechanical Properties

#### 2.12.1. Hardness

The hardness tester (VEB, Werkstoffprűfmaschinen, Leipzig, Germany) was used to determine the ball indentation hardness (*HB*). The procedure was carried out according to the ISO 2039-1:2001 standard [47] on disc-like specimens with dimensions of 40 mm × 4 mm (diameter × thickness).

*HB* was calculated using the following equation:

(5) $$HB\;\left(MPa\right)=\frac{F_{m}\frac{0.21}{\left(h-h_{r}\right)+0.21}}{\pi dh_{r}}$$

The variables in the equation are defined as follows:

*F_m_*—the test load;
*d*—the ball intender diameter (*d* = 5 mm);
*h*—the immersion depth;
*h_r_*—the reduced immersion depth (*h_r_* = 0.25 mm).

#### 2.12.2. Flexural Properties

The universal testing machine (Z020, Zwick, Ulm, Germany) was used to determine the flexural strength (*FS*) and flexural modulus (*E*). The procedure was carried out according to the ISO 178:2019 standard [48] on bar specimens with dimensions of 64 mm × 10 mm × 3.3 mm (length × width × thickness).

*FS* and *E* were calculated using the following equations:

(6) $$FS\;\left(MPa\right)=\frac{3Pl}{2bd^{2}},$$

(7) $$E\;\left(MPa\right)=\frac{P_{1}l^{3}}{4bd^{3}\delta }$$

The variables in the equation are defined as follows:

P_1_—the load at the selected point of the elastic region of the stress–strain plot;
*P*—the maximum load;
*l*—the support span;
*b*—the sample width;
*d*—the sample thickness;
*δ*—the deflection of the sample at *P*_1_.

### 2.13. Antibacterial Properties

The antibacterial properties were characterized by the minimum bactericidal concentration (*MBC*) and minimum inhibitory concentration (*MIC*) against *S. aureus* (ATCC 25923) and *E. coli* (ATCC 25922). The tryptic soy broth (TSB) nutritious medium was used for the bacteria cultivation, which was carried out at 37 °C for 18 h in an incubator (ILW 53, POL-EKO, Wodzisław Śląski, Poland). The experiments were carried out on polymer powders sieved to a grain diameter of less than 25 µm.

A series of six copolymer suspensions in TSB were prepared with concentrations of 50, 25, 12.5, 6.25, 3.125, and 1.563 mg/mL. Next, 20 µL of *S. aureus* and *E. coli* bacterial suspensions in TSB (~5 × 10^8^ CFU/mL) were introduced into the copolymer suspensions, vortexed (10 s, 2000 rpm), and incubated at 37 °C for 18 h. Suspensions were then vortexed again (10 s, 2000 rpm), and 100 µL of each was applied on an agar plate (Müller-Hinton agar, Diag-Med, Raszyn, Poland). The agar plates were incubated at 37 °C for 18 h. The number of bacterial colonies was qualitatively assessed by visual comparison to the control. The lowest concentration at which no bacterial colonies were observed on agar plates was taken as the *MBC*. The lowest concentration at which the bacteria growth was reduced was taken as the *MIC*.

### 2.14. Statistical Analysis

The software (Statistica 13.3, TIBCO Software Inc., Palo Alto, CA, USA) was used to perform the non-parametric Wilcoxon test with a significance level (p) of 0.05. The statistical significance of the results was determined for five samples. The results were expressed as the average value (*AV*) and corresponding standard deviation (*SD*).

## 3. Results

### 3.1. Monomer Synthesis and Characterization

As part of this work, two QAUDMA monomers, QA10+TMXDI and QA12+TMXDI, were obtained. To achieve this goal, a procedure developed for the QAn+TMDI monomer synthesis was adopted [37].

**Figure 1:**
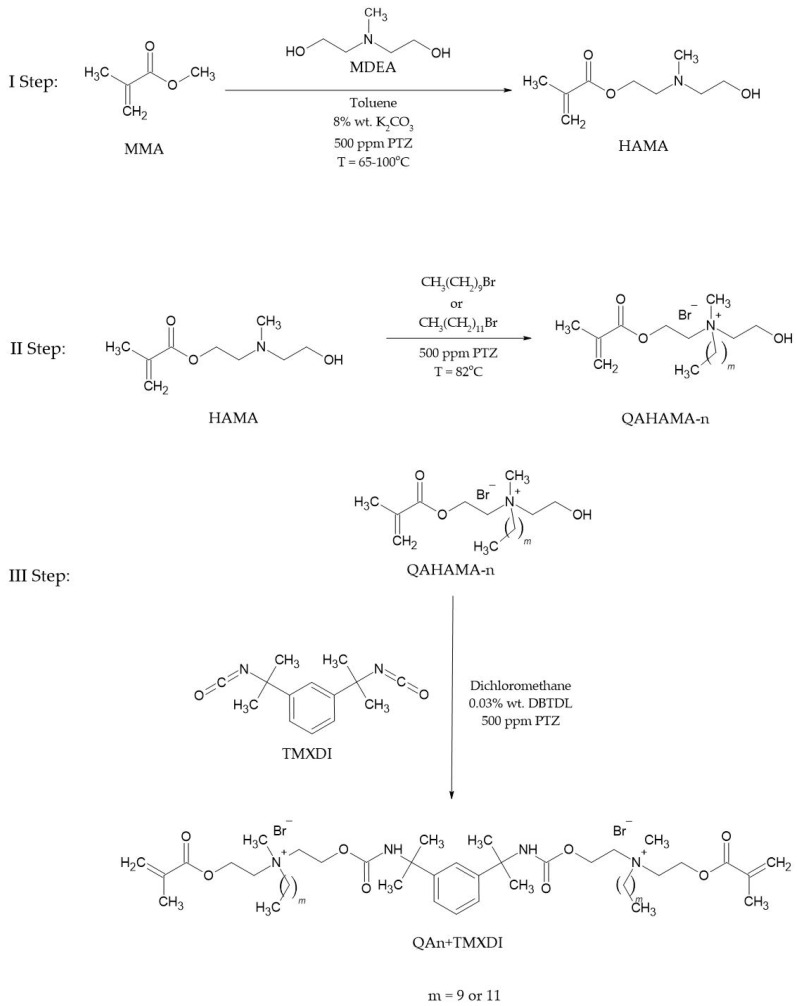
The synthesis route of QAUDMA monomers, namely QA10+TMXDI and QA12+TMXDI.

The synthesis route of QA10+TMXDI and QA12+TMXDI included three stages (Figure 1):

- The transesterification of MMA with MDEA that led to HAMA formation. It was conducted with the MMA excess and K_2_CO_3_ catalyst;
- The Menshutkin reaction by the reaction of HAMA with DB and DDB resulted in a conversion of the HAMA tertiary amine group into a quaternary ammonium group in QAHAMA-n. QAHAMA-10 and QAHAMA-12 were achieved, respectively;
- The addition reaction of QAHAMA-n and TMXDI was conducted with the DBTDL catalyst and resulted in the formation of QAUDMAs: QA10+TMXDI and QA12+TMXDI;
- The chemical structure of the obtained monomers was confirmed using spectroscopic methods, including ^1^H NMR, ^13^C NMR, and FTIR.

**Figure 2:**
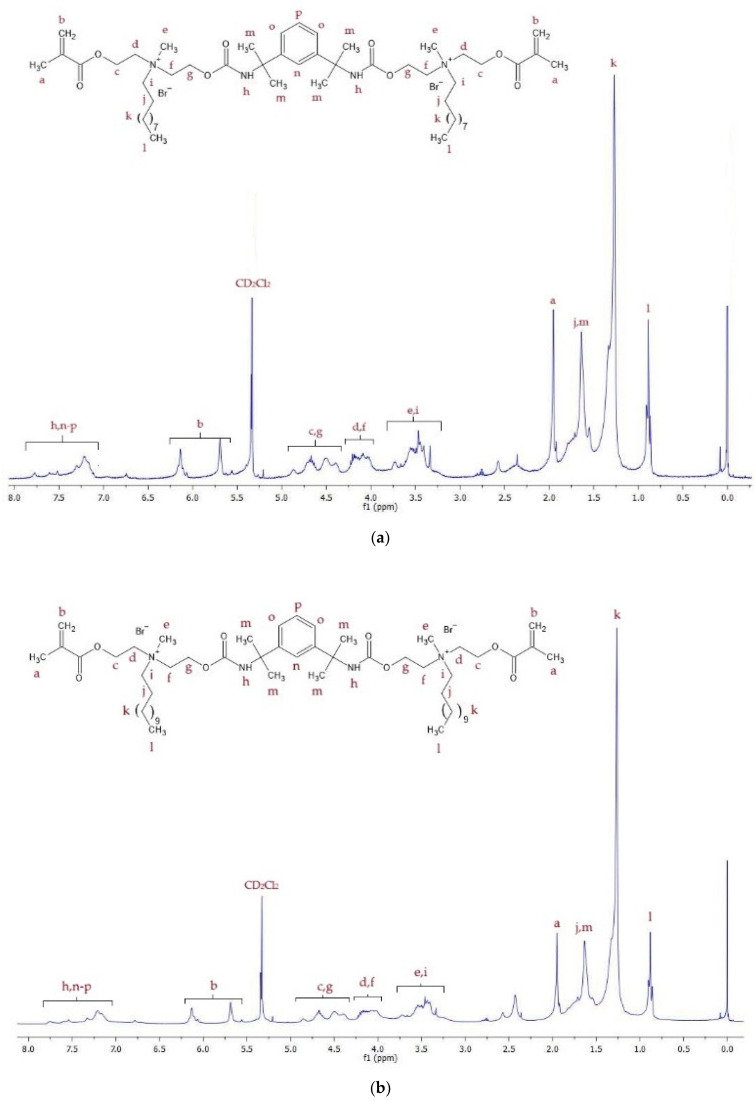
^1^H NMR spectra of monomers: (**a**) QA10+TMXDI; (**b**) QA12+TMXDI.

Figure 2 shows the ^1^H NMR spectra of the QA10+TMXDI and QA12+TMXDI monomers. Table 1 lists the proton signals present in the spectra with their assignment to chemical groups.

**Figure 3:**
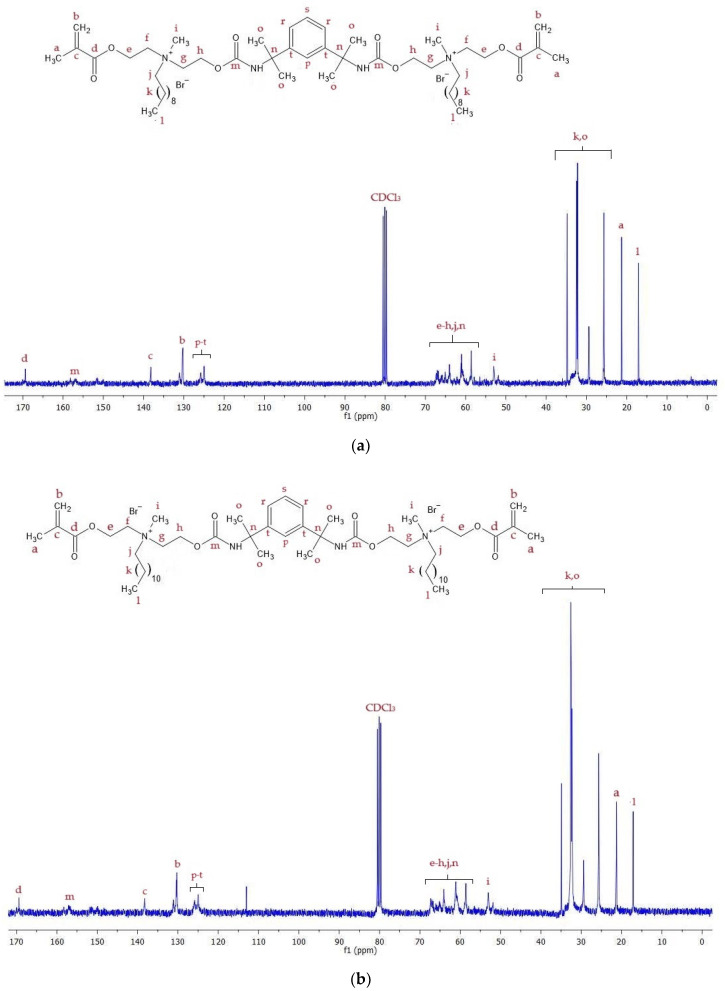
^13^C NMR spectra of monomers: (**a**) QA10+TMXDI; (**b**) QA12+TMXDI.

Figure 3 shows the ^13^C NMR spectra of the QA10+TMXDI and QA12+TMXDI monomers. Table 2 lists carbon atom signals present in the spectra with their assignment to chemical groups.

**Figure 4:**
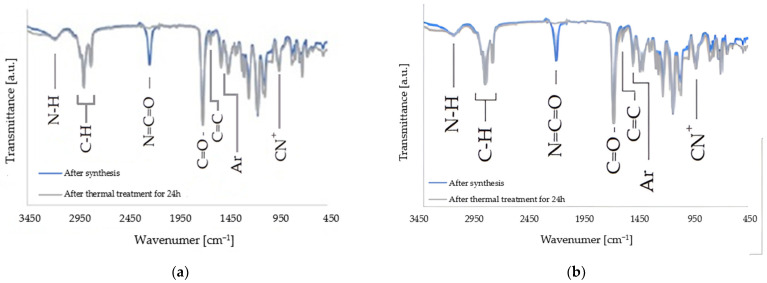
FTIR spectra of the achieved QAUDMA monomers: (**a**) QA10+TMXDI; (**b**) QA12+TMXDI.

Figure 4 shows the FTIR spectra of the QA10+TMXDI and QA12+TMXDI monomers. Table 3 lists of absorption bands present in the spectra with their assignment to chemical groups.

The QA10+TMXDI and QA12+TMXDI monomers were characterized by their molecular weight (*MW*), concentration of double bonds (*x_DB_*), refractive index (*RI*), and density (*d_m_*). Table 4 presents the results for the experimental monomers and three commercial dental dimethacrylate monomers (Bis-GMA, UDMA, and TEGDMA), for comparison.

The molecular weight (*MW*) of QA10+TMXDI was 1061 g/mol and QA12+TMXDI had a *MW* that was 5% higher. Compared to Bis-GMA, UDMA, and TEGDMA, QA10+TMXDI as well as QA12+TMXDI had a higher *MW*, on average by 53, 57, and 74%, respectively. The *MW* was converted to the concentration of double bonds (*x_DB_*). The *x_DB_* of QA10+TMXDI was 1.89 mol/kg and QA12+TMXDI had a *x_DB_* that was 5% lower. Compared to Bis-GMA, UDMA, and TEGDMA, QA10+TMXDI as well as QA12+TMXDI had a lower *x_DB_*, on average by 53, 57, and 74%, respectively.

The refractive index (*RI*) of QA10+TMXDI was 1.5230 and QA12+TMXDI had a *RI* that was 0.6% lower. This difference did not have statistical significance. Compared to Bis-GMA, QA10+TMXDI as well as QA12+TMXDI had a lower *RI*, on average by 2%. Compared to UDMA and TEGDMA, QA10+TMXDI as well as QA12+TMXDI had a higher *RI*, on average by 4 and 3%, respectively.

The density (*d_m_*) of QA10+TMXDI was 1.16 g/cm^3^ and QA12+TMXDI had a *d_m_* that was 15% higher. This difference was statistically significant. Compared to Bis-GMA, UDMA, and TEGDMA, QA10+TMXDI as well as QA12+TMXDI had a higher *d_m_*. In the case of QA10+TMXDI, these differences were 1, 7, and 9%, respectively, for Bis-GMA, UDMA, and TEGDMA. In the case of QA12+TMXDI, these differences were greater and equaled 16, 22, and 25%, respectively, for Bis-GMA, UDMA, and TEGDMA.

### 3.2. Characterization of Monomer Compositions

The obtained QA10+TMXDI and QA12+TMXDI monomers were used to prepare compositions with commercial DMA monomers. QAUDMA 20 wt.% was mixed with UDMA 20 wt.%, Bis-GMA 40 wt.%, and TEGDMA 20 wt.%. For comparison purposes, the UDMA 40 wt.%, Bis-GMA 40 wt.%, and TEGDMA 20 wt.% mixture was prepared. Table 5 shows the names, weight, and molar ratios of the prepared monomer compositions.

The 20(QA10+TMXDI)_m_ and 20(QA12+TMXDI)_m_ experimental monomer compositions and 40(UDMA)_m_ reference monomer composition were characterized by their molecular weight (*MW*), concentration of double bonds (*x_DB_*), viscosity (*η*), refractive index (*RI*), and density (*d_m_*). Table 6 lists the values of these parameters.

The *MW* of 20(QA10+TMXDI)_m_ was 476 g/mol and 20(QA12+TMXDI)_m_ had a *MW* that was 1% higher. Compared to 40(UDMA)_m_, 20(QA10+TMXDI)_m_ as well as 20(QA12+TMXDI)_m_ had a higher *MW*, by 10 and 11%, respectively. The *MW* was converted to the concentration of double bonds in the monomer compositions. The *x_DB_* of 20(QA10+TMXDI)_m_ was 4.20 mol/kg and 20(QA12+TMXDI)_m_ had a *x_DB_* that was 1% lower. Compared to 40(UDMA)_m_, 20(QA10+TMXDI)_m_ as well as 20(QA12+TMXDI)_m_ had a lower *x_DB_*, by 10 and 11%, respectively.

The *η* of 20(QA10+TMXDI)_m_ was 3.79 Pa∙s and 20(QA12+TMXDI)_m_ had a 15% lower *η*. Compared to 40(UDMA)_m_, 20(QA10+TMXDI)_m_ had a higher *η*, whereas 20(QA12+TMXDI)_m_ had a lower *η*, by 7 and 9%, respectively. All these differences did not have statistical significance.

The *RI* of 20(QA10+TMXDI)_m_ was 1.5101 and 20(QA12+TMXDI)_m_ had a *RI* that was 0.05% higher. Compared to 40(UDMA)_m_, 20(QA10+TMXDI)_m_ as well as 20(QA12+TMXDI)_m_ had a higher *RI*, by 0.30 and 0.35%. All these differences did not have statistical significance.

The *d_m_* of 20(QA10+TMXDI)_m_ was 1.13 g/cm^3^ and 20(QA12+TMXDI)_m_ had a *d_m_* that was 2% lower. This difference was statistically significant. Compared to 40(UDMA)_m_, 20(QA10+TMXDI)_m_ had a *d_m_* that was 2% lower, and this difference was statistically significant, whereas 20(QA12+TMXDI)_m_ had the same *d_m_*, and this result did not have statistical significance.

### 3.3. Characterization of Copolymers

20(QA10+TMXDI)_m_, 20(QA12+TMXDI)_m,_ and 40(UDMA)_m_ were subjected to photopolymerization, utilizing an initiation system consisting of CQ and DMAEMA, which is typical for the DCRM photocuring. The structure of the obtained copolymers was characterized by the degree of conversion (*DC*). The physical properties were characterized by determining the polymerization shrinkage (*S*), glass transition temperature (*T_g_*), water sorption (*WS*), water solubility (*SL*), and water contact angle (*WCA*). The mechanical properties were characterized by determining the hardness (*HB*), flexural modulus (*E*), and flexural strength (*FS*). The copolymers were also tested for antibacterial properties by determining the minimum bactericidal concentration (*MBC*) and minimum inhibitory concentration (*MIC*) against *S. aureus* (ATCC 25923) and *E. coli* (ATCC 25922).

#### 3.3.1. Degree of Conversion (*DC*)

**Figure 5:**
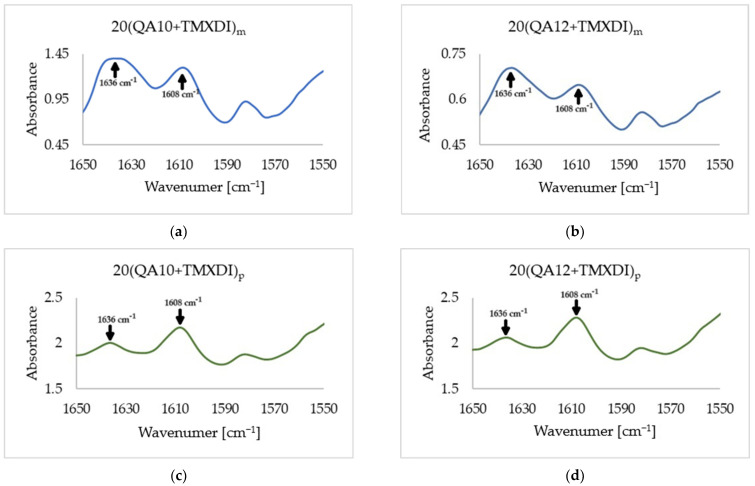
FTIR spectra showing the disappearance of the absorption band of the C=C stretching vibrations at 1636 cm^−1^ due to polymerization, and the absorption band of the aromatic stretching skeletal vibrations at 1608 cm^−1^, which was used as an internal standard: (**a**) 20(QA10+TMXDI)_m_; (**b**) 20(QA12+TMXDI)_m_; (**c**) 20(QA10+TMXDI)_p_; and (**d**) 20(QA12+TMXDI)_p_.

The *DC* was determined utilizing the FTIR spectroscopy and internal standard method. As the tested systems comprised the TMXDI moiety, it was possible to use the aromatic skeletal vibration band as a standard (Figure 5). This is the most widely used method in the *DC* characterization of poly(dimethacrylate)s because this band does not change its specificity due to polymerization [49]. Table 7 shows the *DC* in the copolymers tested.

The *DC* in 20(QA10+TMXDI)_p_ was 0.57 and 20(QA12+TMXDI)_p_ had a *DC* that was 21% higher. This difference was statistically significant. Compared to 40(UDMA)_p_, 20(QA10+TMXDI)_p_ as well as 20(QA12+TMXDI)_p_ had a higher *DC*, by 6 and 28%, respectively. The difference for 20(QA10+TMXDI)_p_ was statistically insignificant, whereas that for 20(QA12+TMXDI)_p_ was statistically significant.

#### 3.3.2. Physical Properties

Table 8 shows the results obtained for the *d_p_*, *S*, and *T_g_* of the copolymers tested. The DSC thermograms of the copolymers are presented in Supplementary Materials in Figure S1.

The *d_p_* of 20(QA10+TMXDI)_p_ was 1.21 g/cm^3^ and 20(QA12+TMXDI)_p_ had a *d_p_* that was 1% lower. Compared to 40(UDMA)_p_, 20(QA10+TMXDI)_p_ had a higher *d_p_*, by 1%, whereas 20(QA12+TMXDI)_p_ had the same *d_p_*. All the results for the *d_p_* were statistically insignificant.

The *S* of 20(QA10+TMXDI)_p_ was 7.04% and the *S* of 20(QA12+TMXDI)_p_ was 10% higher. Compared to 40(UDMA)_p_, 20(QA10+TMXDI)_p_ had a lower *S*, whereas 20(QA12+TMXDI)_p_ had a higher *S*, by 5 and 4%, respectively. All these differences were statistically significant.

The *T_g_* of 20(QA10+TMXDI)_p_ was 53.46 °C and 20(QA12+TMXDI)_p_ had a *T_g_* that was 13% higher. This difference was statistically significant. Compared to 40(UDMA)_p_, 20(QA10+TMXDI)_p_ as well as 20(QA12+TMXDI)_p_ had a lower *T_g_*, by 15 and 4%, respectively. The difference for 20(QA10+TMXDI)_p_ was statistically significant. The other copolymer did not have statistical significance.

Table 9 shows the results obtained for the *WS*, *SL*, and WCA of the tested copolymers. The goniometry camera images of the deionized water droplets on the surfaces of the copolymers are presented in Supplementary Materials in Figure S2.

The *WS* of 20(QA10+TMXDI)_p_ was 10.43 µg/mm^3^ and 20(QA12+TMXDI)_p_ had a *WS* that was 1% higher. This difference was statistically insignificant. Compared to 40(UDMA)_p_, 20(QA10+TMXDI)_p_ as well as 20(QA12+TMXDI)_p_ had a higher *WS*, by 96 and 95%, respectively. These differences were statistically significant.

The *SL* of 20(QA10+TMXDI)_p_ was 2.18 µg/mm^3^ and 20(QA12+TMXDI)_p_ had a *SL* that was 13% higher. Compared to 40(UDMA)_p_, 20(QA10+TMXDI)_p_ as well as 20(QA12+TMXDI)_p_ had a higher *SL*, by 1182 and 1347%, respectively. All these differences were statistically significant.

The *WCA* of 20(QA10+TMXDI)_p_ was 87.03° and 20(QA12+TMXDI)_p_ had a *WCA* that was 5% higher. This difference was statistically insignificant. Compared to 40(UDMA)_p_, 20(QA10+TMXDI)_p_ as well as 20(QA12+TMXDI)_p_ had a higher *WCA*, by 17 and 23%, respectively. These differences were statistically significant.

#### 3.3.3. Mechanical Properties

Table 10 shows the results obtained for the *HB*, *E*, and *FS* of the tested copolymers.

The *HB* of 20(QA10+TMXDI)_p_ was 234.8 MPa and 20(QA12+TMXDI)_p_ had a *HB* that was 17% lower. Compared to 40(UDMA)_p_, 20(QA10+TMXDI)_p_ had a *HB* that was 4% higher, and 20(QA12+TMXDI)_p_ had a *HB* that was 14% lower. All these differences were statistically significant.

The *FS* of 20(QA10+TMXDI)_p_ was 100.63 MPa and 20(QA12+TMXDI)_p_ had a *FS* that was 10% lower. This difference was not statistically significant. Compared to 40(UDMA)_p_, 20(QA10+TMXDI)_p_ and 20(QA12+TMXDI)_p_ had a lower *FS*, by 20 and 28%, respectively. These differences were statistically significant.

The *E* of 20(QA10+TMXDI)_p_ was 3244.9 MPa and 20(QA12+TMXDI)_p_ had a *E* that was 7% lower. This difference was not statistically significant. Compared to 40(UDMA)_p_, 20(QA10+TMXDI)_p_ and 20(QA12+TMXDI)_p_ had a lower *E*, by 10 and 16%, respectively. These differences were statistically significant.

#### 3.3.4. Antibacterial Properties

Table 11 shows the results obtained for the *MIC* and *MBC* of the tested copolymers concerning the *S. aureus* (ATCC 25923) and *E. coli* (ATCC 25922) bacteria strains. The results of the experiments are presented in Supplementary Materials in Figures S3–S8.

20(QA10+TMXDI)_p_ had an *MBC* of 12.5 mg/mL, and an *MIC* of 6.25 mg/mL concerning both bacteria tested. 20(QA12+TMXDI)_p_ had an *MBC* as well as *MIC* greater than 50 mg/mL concerning *S. aureus*. The same copolymer showed a lower *MBC* and *MIC* concerning *E. coli*, respectively, 25 and 12.5 mg/mL. 40(UDMA)_p_ had an *MBC* as well as *MIC* greater than 50 mg/mL in each case, i.e., concerning both bacteria strains.

## 4. Discussion

As part of this study, two novel urethane-dimethacrylate monomers with two QA groups were obtained. For this purpose, the methodology that we developed to obtain an analogous series of urethane-dimethacrylate monomers with two QA groups and a core derived from 1,6-diisocyanato-2,2,4-trimethylhexane (TMDI) was adopted (QAn+TMDI, where n describes the number of carbon atoms in the *N*-alkyl substituent) [37]. Qan+TMDIs were obtained in a three-step process in which HAMA, QAHAMA-n, and Qan+TMDI were successively synthesized. In this study, we used TMXDI instead of TMDI. TMXDI is classified as an aliphatic isocyanate, but it has a central 1,3-phenylene ring [50]. The addition reaction of QAHAMA-n and TMXDI resulted in the formation of QA10+TMXDI and QA12+TMXDI (Figure 1).

The spectroscopic analysis confirmed the chemical structure of the obtained monomers. The ^1^H NMR (Figure 2, Table 1), as well as 13C NMR (Figure 3, Table 2) spectra of QA10+TMXDI and QA12+TMXDI, showed the presence of signals coming from the urethane linkage, TMXDI core, methacrylate group, MDEA moiety, and *N*-alkyl substituent. The signal locations of the -CH_3_ and -CH_2_- groups adjacent to the quaternary nitrogen were specific to the QA group neighborhood. Signals derived from the proton and carbon atoms of the -CH_3_ group neighboring to N^+^ were found from 3.30 to 3.77 ppm in the ^1^H NMR spectra and at 53 ppm in the ^13^C NMR spectra. In the ^1^H NMR spectrum of HAMA, that -CH_3_ group gives a singlet at a higher field, i.e., ca. 2.4 ppm. In the ^13^C NMR spectrum of HAMA, a signal derived from the -CH_3_ group adjacent to N^+^ can be also found at a higher field, i.e., ca. 42 ppm [37]). The formation of the urethane linkage was confirmed by the presence of signals of the -NHCOO- proton, present in the range from 7.10 to 7.80 ppm in the ^1^H NMR spectra, and the -NHCOO- carbon atom, present at 157 ppm in ^13^C NMR spectra. The quaternization and formation of the urethane linkage were additionally confirmed from the FTIR analysis. In the FTIR spectra, a band of the C-N^+^ bond vibrations was present at 943 cm^−1^, whereas the C-N bond vibrations of the -NHCOO- linkage resulted in two bands at 3215 and 1529 cm^−1^ (Figure 4, Table 3).

Physicochemical properties QA10+TMXDI and QA12+TMXDI depended on the *N*-alkyl substituent length (Table 4). Its extension with two methylene groups resulted in a 5% increase in the *MW* and *x_DB_*. The *RI* decreased by 0.6% and the *d_m_* increased by 15%.

QA10+TMXDI and QA12+TMXDI had a *MW* of 1061 and 1117 g/mol and a *x_DB_* of 1.89 and 1.79 mol/kg, respectively (Table 4). This showed that the extension of the *N*-alkyl substituent with two methylene groups resulted in a 5% increase in the *MW* and the same decrease in the *x_DB_*. The *Ris* of QA10+TMXDI and QA12+TMXDI were 1.5230 and 1.5138, respectively. This corresponded to the statistically insignificant decrease in the RI of 0.6% when the *N*-alkyl substituent length increased. The *d_m_* increased from 1.16 to 1.33 g/cm^3^ with the increase in the *N*-alkyl substituent length. This was a 15% change, and this had statistical significance. It can be seen that the physicochemical properties of experimental QAUDMAs depended on the *N*-alkyl substituent length. The more noticeable change was observed for density. The increase in density may suggest the tighter packing of the QA12+TMXDI molecule than that of QA10+TMXDI, regardless of whether it had longer *N*-alkyl substituents.

Compared to Bis-GMA, UDMA, and TEGDMA, QA10+TMXDI and QA12+TMXDI had a higher *MW* (Table 4). The *MW*s of QA10+TMXDI and QA12+TMXDI were approximately more than twice as much as Bis-GMA (512 g/mol) and UDMA (470 g/mol), and almost four times as much as TEGDMA (286 g/mol). This resulted in a lower *x_DB_* of QA10+TMXDI (1.89 mol/kg) and QA12+TMXDI (1.79 mol/kg) compared to dental DMAs (3.90, 4.25, and 6.99 mol/kg, respectively, for Bis-GMA, UDMA, and TEGDMA [37]).

The light-bending ability of QA10+TMXDI and QA12+TMXDI was adequate for a DCRM matrix. Their *Ris* (respectively, 1.5230 and 1.5138) were very close to that of dental DMAs (1.5493, 1.4614, and 1.4852, respectively, for Bis-GMA, UDMA, and TEGDMA [37]) and were within the range of 1.46 to 1.55, which is recommended for dental systems [51,52].

The densities of QA10+TMXDI (1.16 g/cm^3^) and QA12+TMXDI (1.33 g/cm^3^) were higher than those of Bis-GMA, UDMA, and TEGDMA (1.15, 1.09, and 1.07 g/cm^3^, respectively [37]). If compared to fully aliphatic UDMA and TEGDMA, it is understandable that the synthesized QAUDMAs had a higher density, as the TMXDI benzene ring is heavy and capable of tight packing [53]. The comparison of the *d_m_* of QA10+TMXDI and QA12+TMXDI to that of Bis-GMA showed that they had higher densities. Listing the important differences in the chemical structure of experimental QAUDMAs and Bis-GMA may be useful in explaining this observation:

- Bis-GMA has a more spacious core comprising two 1,4-phenylene rings, whereas QA10+TMXDI and QA12+TMXDI has one central 1,3-phenylene ring;
- Bis-GMA has shorter aliphatic wings than QA10+TMXDI and QA12+TMXDI;
- Bis-GMA provides a hydroxyl proton donor to the hydrogen bonds, whereas QA10+TMXDI and QA12+TMXD create these bonds involving the urethane proton donor group;
- QA10+TMXDI and QA12+TMXDI have two long *N*-alkyl substituents in the wings.

It can be concluded that the asymmetrical substitution in the central benzene ring and the long *N*-alkyl substituents do not disturb the tight packing of QA10+TMXDI and QA12+TMXDI.

The QA10+TMXDI and QA12+TMXDI monomers were introduced into the common DCRM dental dimethacrylate composition of UDMA 40 wt.%, Bis-GMA 40 wt.%, and TEGDMA 20 wt.%. The idea was to replace half of UDMA with one of the QAUDMAs, i.e., QA10+TMXDI and QA12+TMXDI. Correspondingly, the novel formulations comprised QAUDMA 20 wt.%, UDMA 20 wt.%, Bis-GMA 40 wt.%, and TEGDMA 20 wt.% (Table 5).

Only a negligible effect of the *N*-alkyl substituent on the 20(QA10+TMXDI)_m_ and 20(QA12+TMXDI)_m_ properties was observed (Table 6). The extension of the *N*-alkyl substituents by two methylene groups resulted in a 1% decrease in the *x_DB_*, a 15% decrease in the *η*, a 0.05% increase in the *RI,* and a 2% increase in the *d_m_*. These differences were statistically insignificant, except for the *d_m_*, which was statistically significant. The most notable change occurred in the viscosity. Its decrease can suggest the weakening of intermolecular interactions in 20(QA12+TMXDI)_m_ caused by the *N*-dodecyl substituent [53].

Compared to 40(UDMA)_m_, the experimental compositions had about a 10% higher *MW* and 10% lower *x_DB_*. This suggests that the replacement of half of UDMA with one of QAUDMAs would have a minor impact on a decrease in the theoretical crosslink density of the resulting copolymer [54].

The comparison of 40(UDMA)_m_ and the experimental compositions by the viscosity, refractive index, and density also led to satisfactory conclusions. 40(UDMA)_m_ had the *η* of 3.53 Pa∙s, which was in the range limited by the *η* of 20(QA10+TMXDI)_m_ and 20(QA12+TMXDI)_m_, i.e., 3.79 and 3.22 Pa∙s, respectively. In addition, differences in the *η* between 40(UDMA)_m_ and 20(QA10+TMXDI)_m_ or 20(QA12+TMXDI)_m_ were only 7 and 9%, respectively, and they did not have statistical significance. This showed that the experimental formulations would have flowability similar to the reference formulation, which would be beneficial to the DCRM preparation. The transparency of 20(QA10+TMXDI)_m_ and 20(QA12+TMXDI)_m_ also was suitable for dental materials. Their *Ris* were of 1.5101 and 1.5094, respectively. This matches the *RI* range of 1.46 to 1.55, which is recommended for dental materials [51,52]. In addition, the difference in the *RI* of the experimental compositions and reference composition was statistically insignificant. Finally, the *d_m_* of 20(QA12+TMXDI)_m_ and 40(UDMA)_m_ had the same value of 1.11 g/cm^3^. 20(QA10+TMXDI)_m_ had a *d_m_* that was 2% higher, and that difference was statistically significant.

20(QA10+TMXDI)_m_ and 20(QA12+TMXDI)_m_ were subjected to photopolymerization towards two experimental copolymers 20(QA10+TMXDI)_p_ and 20(QA12+TMXDI)_p_. They were characterized by the *DC* as the fundamental parameter used to characterize the polymer network structure (Table 7). An appropriately high *DC* is responsible for the adequate physical and mechanical properties of the DCRM matrices and the effective functioning of a dental filling [55,56]. The *DC* of novel QAUDMAs increased statistically significantly with the extension of the *N*-alkyl substituent. 20(QA10+TMXDI)_p_ had a *DC* of 0.57 and 20(QA12+TMXDI)_p_ had a *DC* of 0.69. Their *DC*s also were higher than that in 40(UDMA)_p_., which was 0.54. In addition, The *DC*s of 20(QA10+TMXDI)_p_ and 20(QA12+TMXDI)_p_ were higher than 0.55, which is recommended for clinical applications [57]. Thus, we can conclude that polymerization of 20(QA10+TMXDI)_m_ and 20(QA12+TMXDI)_m_ exhibited high efficiency and was not negatively influenced by a central benzene ring (which increases system stiffness [54]) and *N*-alkyl substituents (which increase the distance between polymerizing species [58,59]) present in their molecules.

A significant effect of the *N*-alkyl substituent on the density, volumetric contraction, glass temperature, water sorption, and leachability of 20(QA10+TMXDI)_p_ and 20(QA12+TMXDI)_p_ was not observed (Table 8 and Table 9). 20(QA10+TMXDI)_p_ had a *d_p_* of 1.21 g/cm^3^, *S* of 7.04%, *T_g_* of 53.46 °C, *WS* of 10.43 µg/mm^3^, and *SL* of 2.18 µg/mm^3^. 20(QA12+TMXDI)_p_ had a 1% lower *d_p_*, and this change was statistically insignificant. The *S* and *T_g_* statistically significantly increased, and these changes were 10% and 13%, respectively. The *WS* decreased statistically insignificantly by 1%, whereas the *SL* increased by 13% and this change was statistically significant. Although the decrease in the *WS* was insignificant, the chemical character of the copolymer surface can explain this change. 20(QA12+TMXDI)_p_ was characterized by a *WCA* of 91.30° (Table 9), which corresponds to a hydrophobic surface [60,61]. 20(QA12+TMXDI)_p_ had a statistically insignificantly lower *WS* and *WCA*. However, its *WCA* was 91.30°, which corresponds to a hydrophobic surface [60,61]. An effect of the *N*-alkyl substituent on the *SL* can be attributed to a higher *MW* of QA12+TMXDI compared to that of QA10+TMXDI. It is possible that a smaller number of unreacted monomer molecules leached out from 20(QA12+TMXDI)_p_ because of a higher *DC* compared to 20(QA10+TMXDI)_p_. A similar effect was also observed in other work on the copolymer of Qan+TMDI [39].

The reference copolymer, 40(UDMA)_p_, had a 7.42% *S*, which was within the range limited by the *S* of 20(QA10+TMXDI)_p_ and 20(QA12+TMXDI)_p_ (Table 8). Compared to 40(UDMA)_p_, 20(QA10+TMXDI)_m_ had a 5% lower *S* and 20(QA12+TMXDI)_m_ had a 4% higher *S*, and both changes were statistically significant. The small scale of these differences suggests that the experimental and reference formulations had similar volumetric contractions caused by polymerization. Therefore, the potential DCRM matrices modified with QA10+TMXDI and QA12+TMXDI would probably have a marginal gap similar to common dental dimethacrylate copolymers [62].

The thermal behavior of 20(QA10+TMXDI)_p_ and 20(QA12+TMXDI)_p_ also was appropriate for their application in the potential DCRM matrix. On one hand, the experimental copolymers had a *T_g_* of 53.46 and 60.30 °C, respectively. These *T_g_*s were lower than the *T_g_* of 40(UDMA)_p_ (62.97 °C). The difference in *T_g_*s between the reference copolymer and 20(QA10+TMXDI)_p_ was 15% and was statistically significant, whereas that for 20(QA12+TMXDI)_p_ was 4% and was statistically insignificant (Table 8). However, the *T_g_*s in both cases were higher than 50 °C. This is a highly satisfactory result because it means that the experimental copolymers occur in a glassy state up to a temperature of at least 50 °C. Therefore, we can assume that the experimental copolymers would maintain constant mechanical performance over the entire temperature range in the oral cavity [63].

Compared to 40(UDMA)_p_, which had a *WS* of 5.31 μg/mm^3^ and *SL* of 0.17 μg/mm^3^, the experimental copolymers had *WS*s almost twice as high and *SL*s approximately fourteen times higher (Table 9). These were statistically significant differences, but the values of the *WS* and *SL* were noticeably lower than those defined by ISO 4049, i.e., 40 µg/mm^3^ and 7.5 µg/mm^3^, respectively [46]. A higher *WS* (10.43 μg/mm^3^), which was determined for 20(QA10+TMXDI)_p_, was 26% of the maximum recommended by the ISO standard, and a higher *SL*, which was determined for 20(QA12+TMXDI)_p_ (2.46 μg/mm^3^), was 33% of the maximum recommended by the ISO 4049 standard. This is a highly satisfactory outcome, as the hydrophilic QA groups usually cause an increase in a polymer’s *WS* [39]. The increased *WS* and *SL* of the experimental copolymers showed that the QA groups promote interactions with water molecules, even though the *N*-alkyl substituent decreased the hydrophilicity of the copolymer surface. This was confirmed by the result achieved for 40(UDMA)_p_, which was characterized by the lowest *WCA* (74.07°, Table 9), and thus its surface was the most hydrophilic [60].

The mechanical properties of 20(QA10+TMXDI)_p_ and 20(QA12+TMXDI) _p_ decreased as the *N*-alkyl substituent length increased. The *E*, *FS*, and *HB* for 20(QA10+TMXDI)_p_ were 3244.9 Mpa, 100.6 Mpa, and 234.8 Mpa, respectively. They decreased by 7%, 10%, and 17% for 20(QA12+TMXDI)_p_ (Table 10). Only the result for the *HB* was statistically significant. This led to the conclusion that hardness was the most sensitive to the *N*-alkyl chain extension, as its decline was the most pronounced.

40(UDMA)p exhibited the *E*, *FS*, and *HB* of 3597.7 Mpa, 125.4 Mpa, and 225.9 Mpa, respectively. Its modification by introducing QAUDMA resulted in statistically significant decreases of *E*, by 10 and 16%, and *FS*, by 20 and 28%, respectively, for 20(QA10+TMXDI)_p_ and 20(QA12+TMXDI)_p_. The *HB* of 20(QA10+TMXDI)_p_ increased by 4% and this difference was statistically significant. As the length of the *N*-alkyl substituent increased to twelve carbon atoms, the *HB* decreased statistically significantly by 14%. However, the overall mechanical performance of 20(QA10+TMXDI)_p_ as well as 20(QA12+TMXDI)_p_ was fully satisfactory, as the tested parameters were high.

Finally, the experimental copolymers revealed antibacterial activity against *S. aureus* and *E. coli* (Table 11). 20(QA10+TMXDI)_p_ had a lower *MIC* and *MBC* than 20(QA12+TMXDI)_p_. This means that the extension of the *N*-alkyl substituent in the QAUDMA with two methylene groups decreased the copolymer’s antibacterial activity. If compared to 40(UDMA)_p_, both copolymers had higher antibacterial activity. In addition, intra-species differences were observed for 20(QA12+TMXDI)_p_. If compared to 40(UDMA)_p_, 20(QA12+TMXDI)_p_ showed the same antibacterial activity concerning *S. aureus*, and greater antibacterial activity concerning *E.coli*. 20(QA12+TMXDI)_p_ had a *MBC* 50% lower and *MIC* 75% lower than 40(UDMA)_p_ in tests against *E.coli*. 20(QA10+TMXDI)_p_ showed more significant antibacterial activity and differentiation according to bacterial strain was no longer observed. 20(QA10+TMXDI)_p_ had a 75% lower *MBC* and 87.5% lower *MIC* than 40(UDMA)_p_ in tests against both bacteria strains.

We can additionally characterize the experimental copolymers using a common classification of antimicrobials as bactericidal or bacteriostatic. This classification assumes that if the *MBC* to *MIC* ratio is lower than four, a biocide can be recognized bactericidal, and if the *MBC* to *MIC* ratio is higher than six, a biocide can be recognized bacteriostatic [64]. As this ratio for 20(QA10+TMXDI)_p_ was two, one can assume this copolymer is bactericidal and it may be possible to administer the 20(QA10+TMXDI)_p_ dosages to kill 99.9% of the bacteria.

## 5. Conclusions

The two novel quaternary ammonium urethane-dimethacrylate (QAUDMA) monomers were successfully synthesized from 1,3-bis(1-isocyanato-1-methylethyl)benzene TMXDI) and 2-(methacryloyloxy)ethyl-2-alkylhydroxyethylmethylammonium bromides with the decyl and dodecyl *N*-alkyl substituents, respectively, QA10+TMXDI and QA12+TMXDI. The TMXDI diisocyanate provided an aromatic central ring in the QAUDMA structure.

The achieved QAUDMAs were used as comonomers 20 wt.% in the copolymerization with common dental dimethacrylates, UDMA 20 wt.%, Bis-GMA 40 wt.%, and TEGDMA 20 wt.%. For comparison, the UDMA 40 wt.%, Bis-GMA 40 wt.%, and TEGDMA 20 wt.% representative dental copolymer was also prepared.

The monomer compositions had adequate flowability and transparency required for dental materials.

A comparative analysis of the tested copolymers’ properties showed that their polymerization shrinkage, glass transition temperature, and mechanical properties, were satisfactory and were usually similar to the properties of the reference copolymer. The water sorption and water solubility mostly deteriorated; however, they were satisfactory. Their values were far below the limits pointed at the international standard ISO 4049.

The antibacterial properties of the copolymers were characterized against *S. aureus* (ATCC 25923) and *E. coli* (ATCC 25922). The copolymer comprising QA10+TMXDI showed a significantly higher antibacterial effect than both the copolymer comprising QA12+TMXDI and the reference copolymer, as it had a lower minimum bactericidal concentration (*MBC*) and minimum inhibitory concentration (*MIC*).

This study demonstrated that the introduction of a benzene ring into the QAUDMA structure resulted in copolymers with very good physicochemical and mechanical characteristics and antibacterial activity. However, it was observed that the shorter the *N*-alkyl chain, the higher the antibacterial activity. Therefore, it is worth conducting further research with monomers that have shorter *N*-alkyl substituents. A more in-depth investigation of antibacterial properties should also be carried out for the tested copolymers, as the aim of this work was only to determine whether they have antibacterial activity.

## Figures and Tables

**Table 1:** ^1^H NMR signals of QA10+TMXDI and QA12+TMXDI monomers.

Signal Symbol	Hydrogen Atom	Multiplicity	Number of Protons	Chemical Shift [ppm]
a	CH_3_-C=	s	6	1.95
b	=CH_2_	2 m	4	5.70 and 6.14
c	-O-CH_2_-CH_2_-N^+^-	m	4	4.33–4.91
d	-O-CH_2_-CH_2_-N^+^-	m	4	4.00–4.26
e	-N^+^-CH_3_	s	6	3.30–3.77
f	-O-CH_2_-CH_2_-N^+^-	m	4	4.00–4.26
g	-O-CH_2_-CH_2_-N^+^-	m	4	4.33–4.91
h	-NH-C=O	m	2	7.10–7.80
i	-N^+^-CH_2_-CH_2_-(CH_2_)_7(or 9)_-CH_3_ ^1^	m	4	3.30–3.77
j	-N^+^-CH_2_-CH_2_-(CH_2_)_7(or 9)_-CH_3_ ^1^	m	4	1.49–1.88
k	-N^+^-CH_2_-CH_2_-(CH_2_)_7(or 9)_-CH_3_ ^1^	m	28/36 ^2^	1.16–1.47
l	-N^+^-CH_2_-CH_2_-(CH_2_)_(7 or 9)_-CH_3_ ^1^	m	6	0.82–1.00
m	CH_3_-C-CH_3_	m	12	1.49–1.88
n	-CH- (Ar)	m	1	7.10–7.80
o	-CH- (Ar)	m	2	7.10–7.80
p	-CH- (Ar)	m	1	7.10–7.80

^1^ -N^+^-CH_2_-CH_2_-(CH_2_)_7_-CH_3_ corresponds to QA10+TMXDI, and -N^+^-CH_2_-CH_2_-(CH_2_)_9_-CH_3_ corresponds to QA12+TMXDI. ^2^ 28 corresponds to QA10+TMXDI, and 36 corresponds to QA12+TMXDI.

**Table 2:** ^13^C NMR signals of QA10+TMXDI and QA12+TMXDI monomers.

Signal Symbol	Carbon Atom	Chemical Shift [ppm]
a	CH_3_-C=	21
b	=CH_2_	130
c	CH_3_-C=CH_2_	138
d	-O-C=O	169
e-h	-CH_2_-	59–67
i	-N^+^-CH_3_	53
j	-N^+^-CH_2_-(CH_2_)_8(or 10)_-CH_3_^1^	59–67
k	-N^+^-CH_2_-(CH_2_)_8(or 10)_-CH_3_^1^	26–35
l	-N^+^-CH_2_-(CH_2_)_8 (or 10)_-CH_3_^1^	17
m	-NH-C=O	157
n	-NH-C-	59–67
o	>C-CH_3_	26–35
p-s	-CH= (Ar)	124–126
t	>C= (Ar)	

^1^ -N^+^-CH_2_-(CH_2_)_8_-CH_3_ corresponds to QA10+TMXDI, and -N^+^-CH_2_-(CH_2_)_10_-CH_3_ corresponds to QA12+TMXDI.

**Table 3:** The interpretation of FTIR spectra of QA10+TMXDI and QA12+TMXDI monomers.

Chemical Bond	Intensity ^1^	Wavenumber [cm^−1^]
N-H	w	3215
CH_3_	w	3024
=CH_2_	m	2957
CH_2_, CH_3_	m	2925 and 2855
N=C=O ^2^	s	2257
C=O	s	1714
C=C	w	1638
C=C (Ar)	w	1605
NH (urethane)	m	1529
CH_2_, CH_3_	m	1457
C-N	m	1249 and 1158
C-O-C	m	1092 and 1087
C-N^+^	m	943

^1.^ The intensity of the absorption bands in the FTIR spectra was referred to as strong (s), medium (m), and weak (w). ^2.^ Refers to the isocyanate group absorption band present in the FTIR spectra of the QAUDMA monomers before their thermal treatment.

**Table 4:** Properties of QA10+TMXDI and QA12+TMXDI related to properties of Bis-GMA, UDMA, and TEGDMA. The lowercase letter “a” in the superscripts indicate couples with statistically significant results (*p* ≤ 0.05). The remaining results did not have statistical significance (*p* > 0.05).

Monomer Name	*MW* (g/mol)	*x_DB_* (mol/kg)	*RI*	*d_m_* (g/cm^3^)
			*AV* ^1^	*AV* ^2^
Experimental monomers				
QA10+TMXDI	1061	1.89	1.5230	1.16 ^a^
QA12+TMXDI	1117	1.79	1.5138	1.33 ^a^
Reference monomers				
Bis-GMA	512	3.90	1.5493 ^3^	1.15 ^3^
UDMA	470	4.25	1.4614 ^3^	1.09 ^3^
TEGDMA	286	6.99	1.4852 ^3^	1.07 ^3^

^1^ The *SD* for the *RI* values was 0.0001. ^2^ The *SD* for the *d_m_* values was 0.01. ^3^ Taken from [37].

**Table 5:** Compositions of QAUDMA, Bis-GMA, UDMA, and TEGDMA experimental mixtures related to the Bis-GMA, UDMA, and TEGDMA reference mixture.

Monomer Composition	Sample Composition							
	QAUDMA		UDMA		Bis-GMA		TEGDMA	
	Mass Fraction	Mole Fraction	Mass Fraction	Mole Fraction	Mass Fraction	Mole Fraction	Mass Fraction	Mole Fraction
Experimental compositions								
20(QA10+TMXDI)_m_	0.20	0.09	0.20	0.20	0.40	0.37	0.20	0.34
20(QA12+TMXDI)_m_	0.20	0.09	0.20	0.20	0.40	0.37	0.20	0.34
Reference composition								
40(UDMA)_m_	0.00	0.00	0.40	0.36	0.40	0.34	0.20	0.30

**Table 6:** Properties of 20(QA10+TMXDI)_m_ and 20(QA12+TMXDI)_m_ experimental monomer compositions related to properties of the 40(UDMA)_m_ reference monomer composition. The lowercase letters “a” and “b” in the superscripts indicate couples with statistically significant results (*p* ≤ 0.05). The remaining results did not have statistical significance (*p* > 0.05).

Monomer Composition	*MW* (g/mol)	*x_DB_* (mol/kg)	*η* (Pa∙s)		*RI*	*d_m_* (g/cm^3^)
			*AV*	*SD*	*AV* ^1^	*AV* ^2^
Experimental compositions						
20(QA10+TMXDI)_m_	476	4.20	3.79	0.40	1.5101	1.13 ^a,b^
20(QA12+TMXDI)_m_	481	4.16	3.22	0.30	1.5094	1.11 ^a^
Reference composition						
40(UDMA)_m_	429	4.66	3.53	0.40	1.5049	1.11 ^b^

^1^ The *SD* for the *RI* values always was 0.0001. ^2^ The *SD* for the *d_m_* values was always 0.01.

**Table 7:** The *DC* in copolymers. The lowercase letters “a” and “b” in the superscripts indicate couples with statistically significant results (*p* ≤ 0.05). The remaining results did not have statistical significance (*p* > 0.05).

Copolymer	*DC*	
	*AV*	*SD*
Experimental copolymers		
20(QA10+TMXDI)_p_	0.57 ^a^	0.05
20(QA12+TMXDI)_p_	0.69 ^a,b^	0.06
Reference copolymer		
40(UDMA)_p_	0.54 ^b^	0.04

**Table 8:** The physical properties of copolymers: density (*d_p_*), polymerization shrinkage (*S*), and glass transition temperature (*T_g_*). The lowercase letters “a”–“c” in the superscripts indicate couples with statistically significant results (*p* ≤ 0.05). The remaining results did not have statistical significance (*p* > 0.05).

Copolymer	*d_p_* (g/cm^3^)		*S* (%)		*T_g_* (°C)	
	*AV*	*SD*	*AV*	*SD*	*AV*	*SD*
Experimental copolymers						
20(QA10+TMXDI)_p_	1.21	0.01	7.04 ^a,b^	0.83	53.46 ^a,b^	3.27
20(QA12+TMXDI)_p_	1.20	0.01	7.72 ^a,c^	1.17	60.30 ^a^	4.34
Reference copolymer						
40(UDMA)_p_	1.20	0.01	7.42 ^b,c^	1.08	62.97 ^b^	4.52

**Table 9:** The physical properties characterizing copolymers’ interaction with water: water sorption (*WS*), water leachability (*SL*), and water contact angle (*WCA*). The lowercase letters “a”–“c” in the superscripts indicate couples with statistically significant results (*p* ≤ 0.05). The remaining results did not have statistical significance (*p* > 0.05).

Copolymer	*WS* (μg/mm^3^)		*SL* (μg/mm^3^)		*WCA* (◦)	
	*AV*	*SD*	*AV*	*SD*	*AV*	*SD*
Experimental copolymers						
20(QA10+TMXDI)_p_	10.43 ^a^	0.42	2.18 ^a,b^	0.06	87.03 ^a^	3.07
20(QA12+TMXDI)_p_	10.35 ^b^	0.23	2.46 ^a,c^	0.25	91.30 ^b^	4.82
Reference copolymer						
40(UDMA)_p_	5.31 ^a,b^	0.36	0.17 ^b,c^	0.09	74.07 ^a,b^	2.24

**Table 10:** The mechanical properties of copolymers: hardness (*HB*), flexural modulus (*E*), and flexural strength (*FS*). The lowercase letters “a”–“c” in the superscripts indicate couples with statistically significant results (*p* ≤ 0.05). The remaining results did not have statistical significance (*p* > 0.05).

**Copolymer**	***HB* (MPa)**		***FS* (MPa)**		***E* (MPa)**	
	***AV***	***SD***	***AV***	***SD***	***AV***	***SD***
Experimental copolymers						
20(QA10+TMXDI)_p_	234.8 ^a,b^	8.7	100.6 ^a^	11.7	3244.9 ^a^	114.1
20(QA12+TMXDI)_p_	194.6 ^a,c^	26.5	90.3 ^b^	6.1	3020.6 ^b^	198.9
Reference copolymer						
40(UDMA)_p_	225.9 ^b,c^	19.4	125.4 ^a,b^	13.0	3597.7 ^a,b^	154.4

**Table 11:** The antibacterial properties of copolymers against *S. aureus* (ATCC 25923) and *E. coli* (ATCC 25922): minimum bactericidal concentration (*MBC*) and minimum inhibitory concentration (*MIC*).

Copolymer	*MBC* [mg/mL]		*MIC* [mg/mL]	
	*S. aureus * (ATCC 25923)	*E. coli* (ATCC 25922)	*S. aureus * (ATCC 25923)	*E. coli* (ATCC 25922)
Experimental copolymers				
20(QA10+TMXDI)_p_	12.5	12.5	6.25	6.25
20(QA12+TMXDI)_p_	>50	25	>50	12.5
Reference copolymer				
40(UDMA)_p_	>50	>50	>50	>50
Control				
Sterile Water	-	-	-	

## Data Availability

Data are contained within the article and supplementary materials.

## Supplementary Materials

The following supporting information can be downloaded at: https://www.mdpi.com/article/10.3390/ma17020298/s1, Figure S1: DSC thermograms of copolymers: (a) 20(QA10+TMXDI)_p_; (b) 20(QA12+TMXDI) _p_; (c) 40(UDMA) _p_, Figure S2: The goniometry camera images of deionized water droplets on the surfaces of copolymers: (a) 20(QA10+TMXDI)_p_; (b) 20(QA12+TMXDI) _p_; (c) 40(UDMA) _p_, Figure S3: The results of antibacterial activity tests of 20(QA10+TMXDI)_p_ against *S. aureus* (ATCC 25923), Figure S4: The results of antibacterial activity tests of 20(QA12+TMXDI)_p_ against *S. aureus* (ATCC 25923), Figure S5: The results of antibacterial activity tests of 40(UDMA)_p_ against *S. aureus* (ATCC 25923), Figure S6: The results of antibacterial activity tests of 20(QA10+TMXDI)_p_ against *E. coli* (ATCC 25922), Figure S7: The results of antibacterial activity tests of 20(QA12+TMXDI)_p_ against *E. coli* (ATCC 25922), Figure S8: The results of antibacterial activity tests of 40(UDMA)_p_ against *E. coli* (ATCC 25922).
