# Supplementary material for: Chemical Modification of Dental Dimethacrylate Copolymer with Tetramethylxylylene Diisocyanate-Based Quaternary Ammonium Urethane-Dimethacrylates—Physicochemical, Mechanical, and Antibacterial Properties
Source: Materials (Basel). 2024 Jan 7;17(2):298. doi: 10.3390/ma17020298 (PMC10817292; doi:10.3390/ma17020298)
Supplement: Supplementary file 1 [file materials-17-00298-s001.zip › materials-2806268-supplementary.pdf]

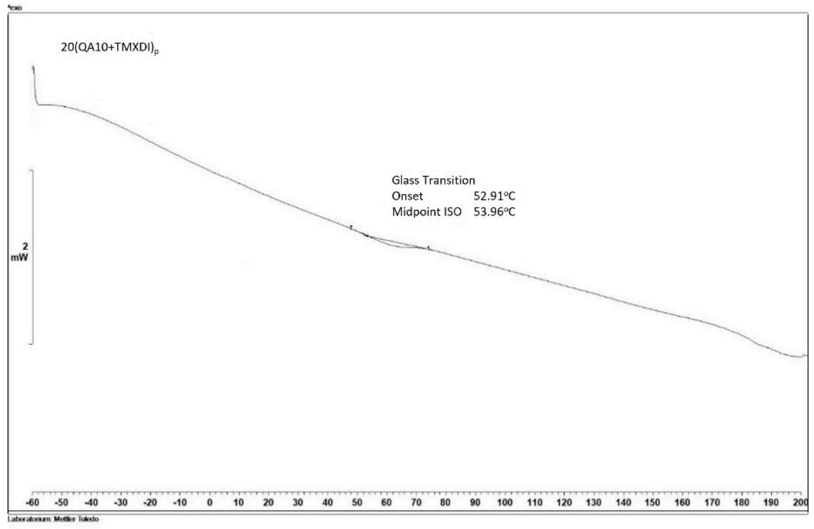

(**a**)

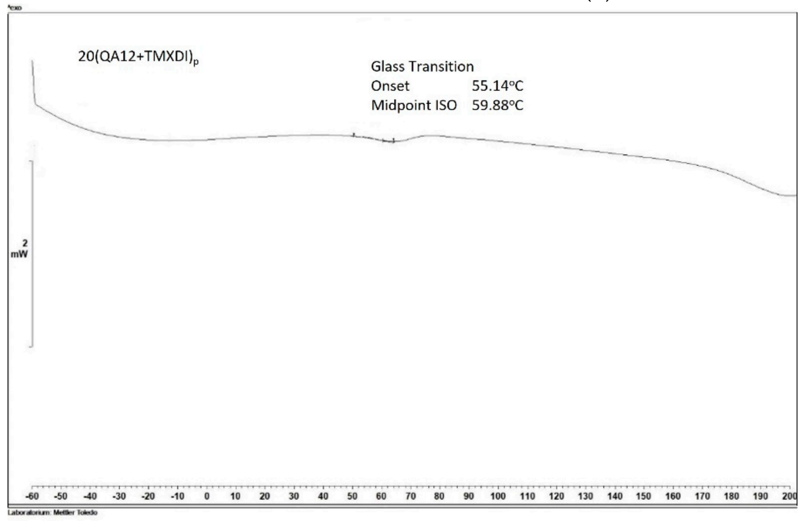

(**b**)

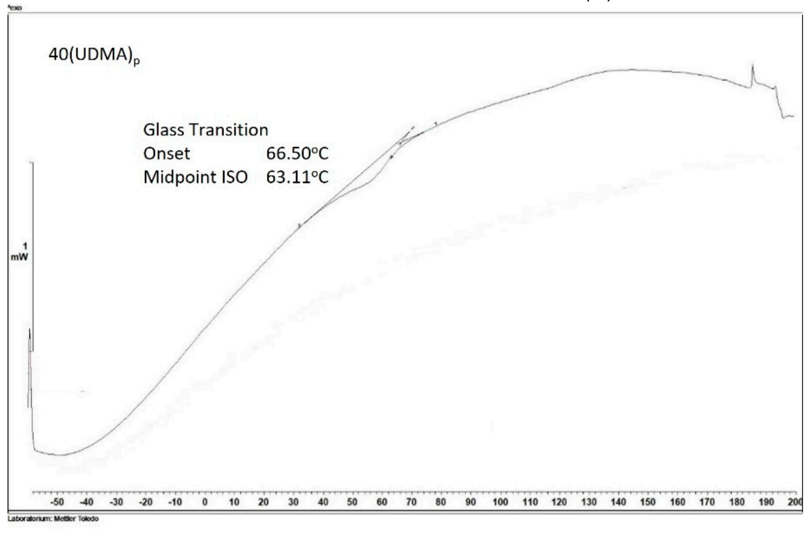

(c)

Figure S1. DSC thermograms of copolymers: (**a**) $20(QA10+TMXDI)_P$; (**b**) $20(QA12+TMXDI)_P$; (**c**) $40(UDMA)_P$.

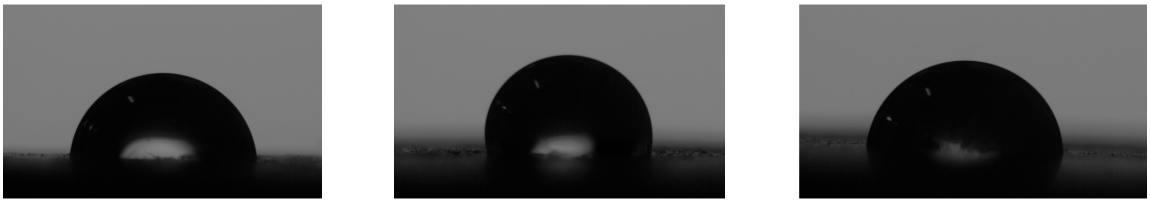

(**a**) (**b**) (**c**)

Figure S2. The goniometry camera images of deionized water droplets on the surfaces of copolymers: (**a**) 20(QA10+TMXDI)$_P$; (**b**) 20(QA12+TMXDI)$_P$; (**c**) 40(UDMA)$_P$.

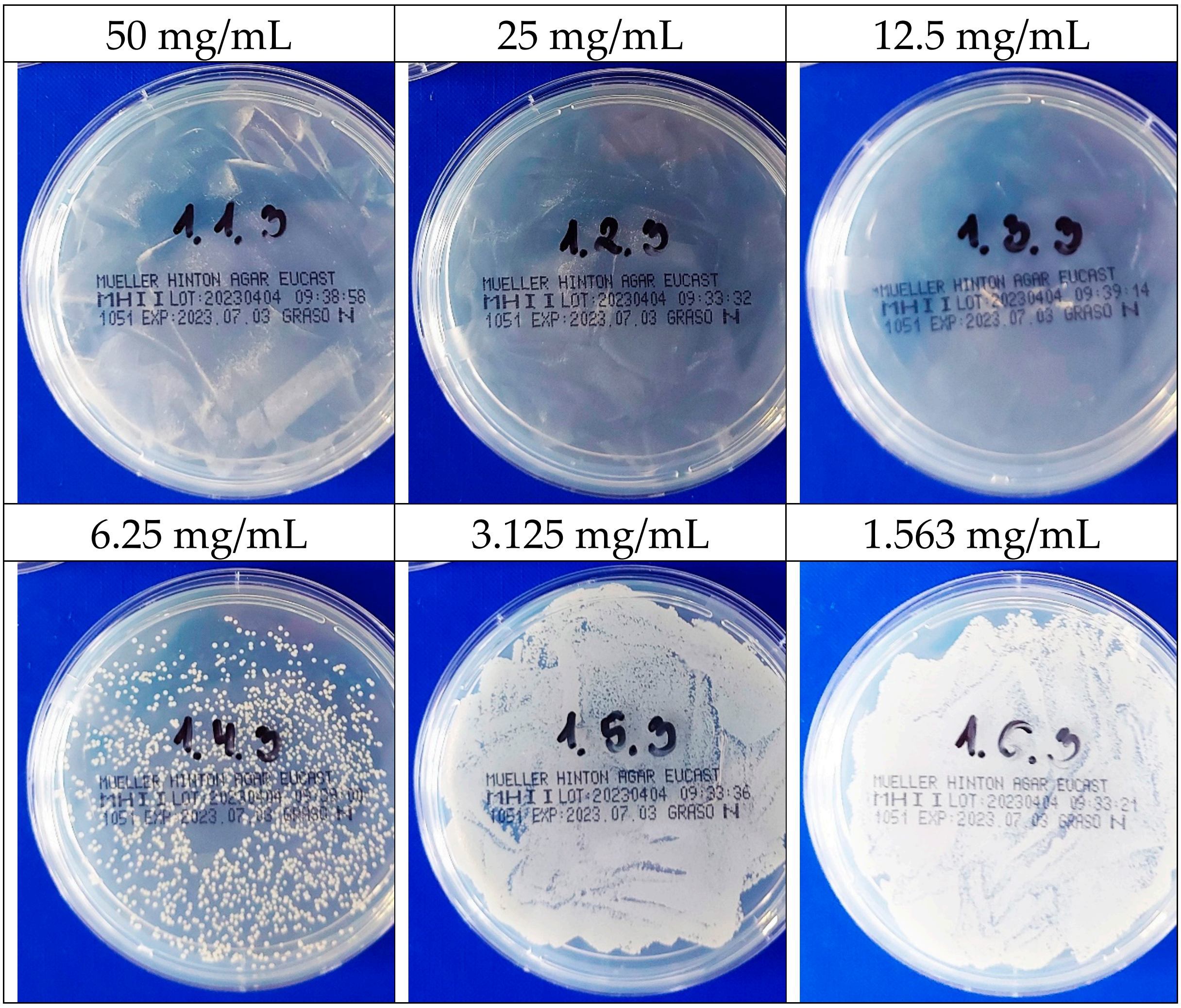

Figure S3. The results of antibacterial activity tests of 20(QA10+TMXDI)$_P$ against *S. aureus* (ATCC 25923).

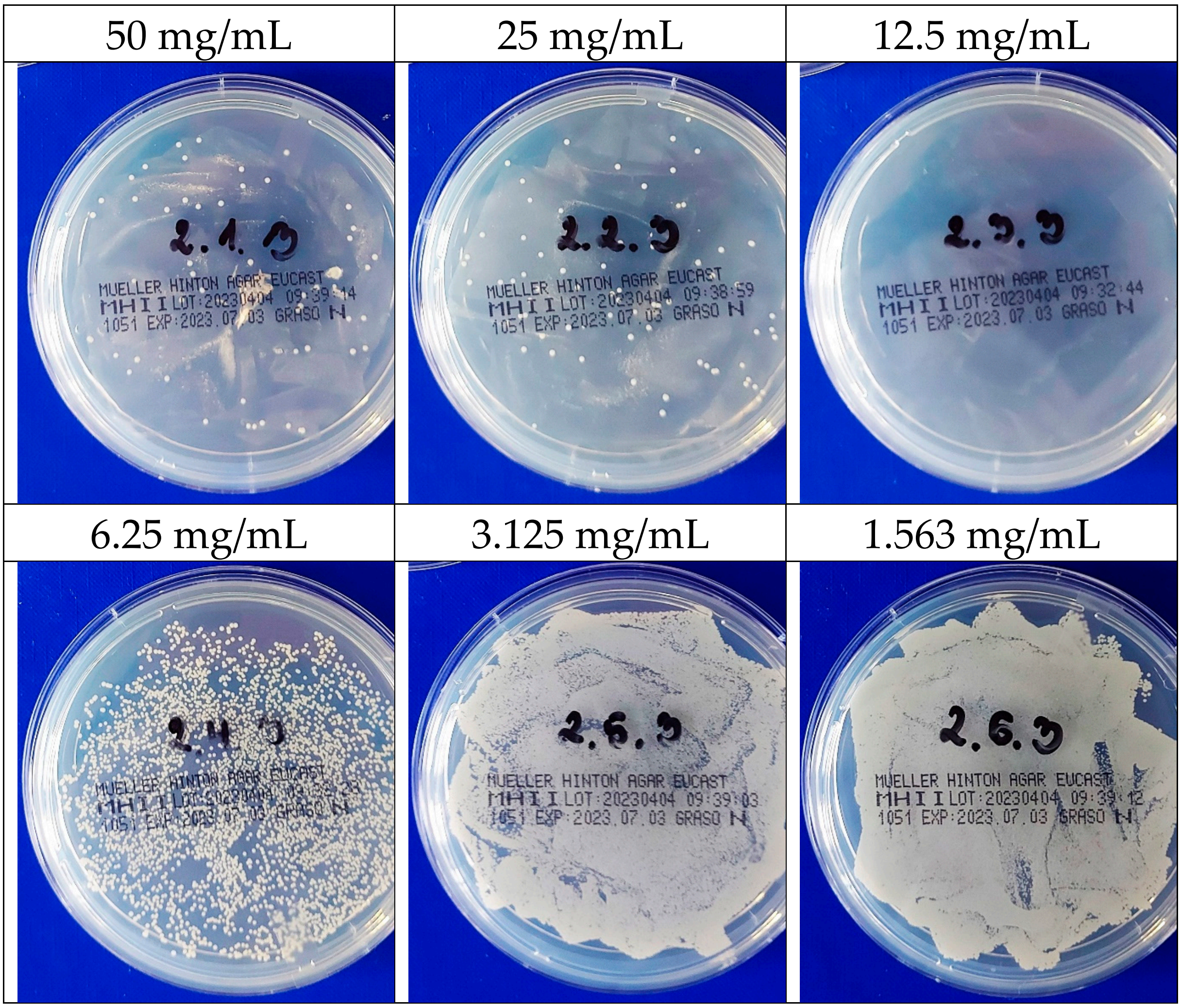

Figure S4. The results of antibacterial activity tests of 20(QA12+TMXDI)$_P$ against *S. aureus* (ATCC 25923).

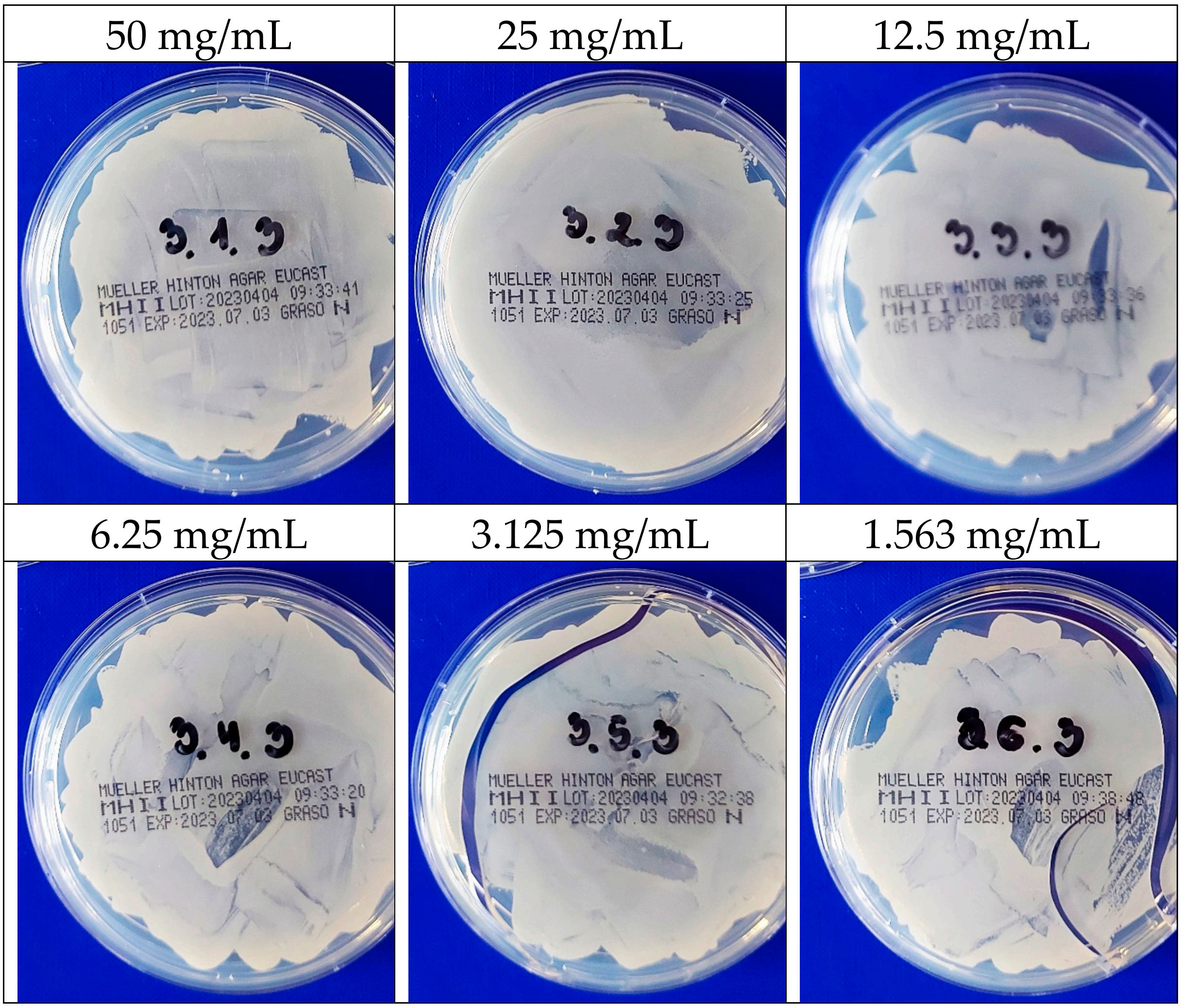

Figure S5. The results of antibacterial activity tests of 40(UDMA)$_P$ against *S. aureus* (ATCC 25923).

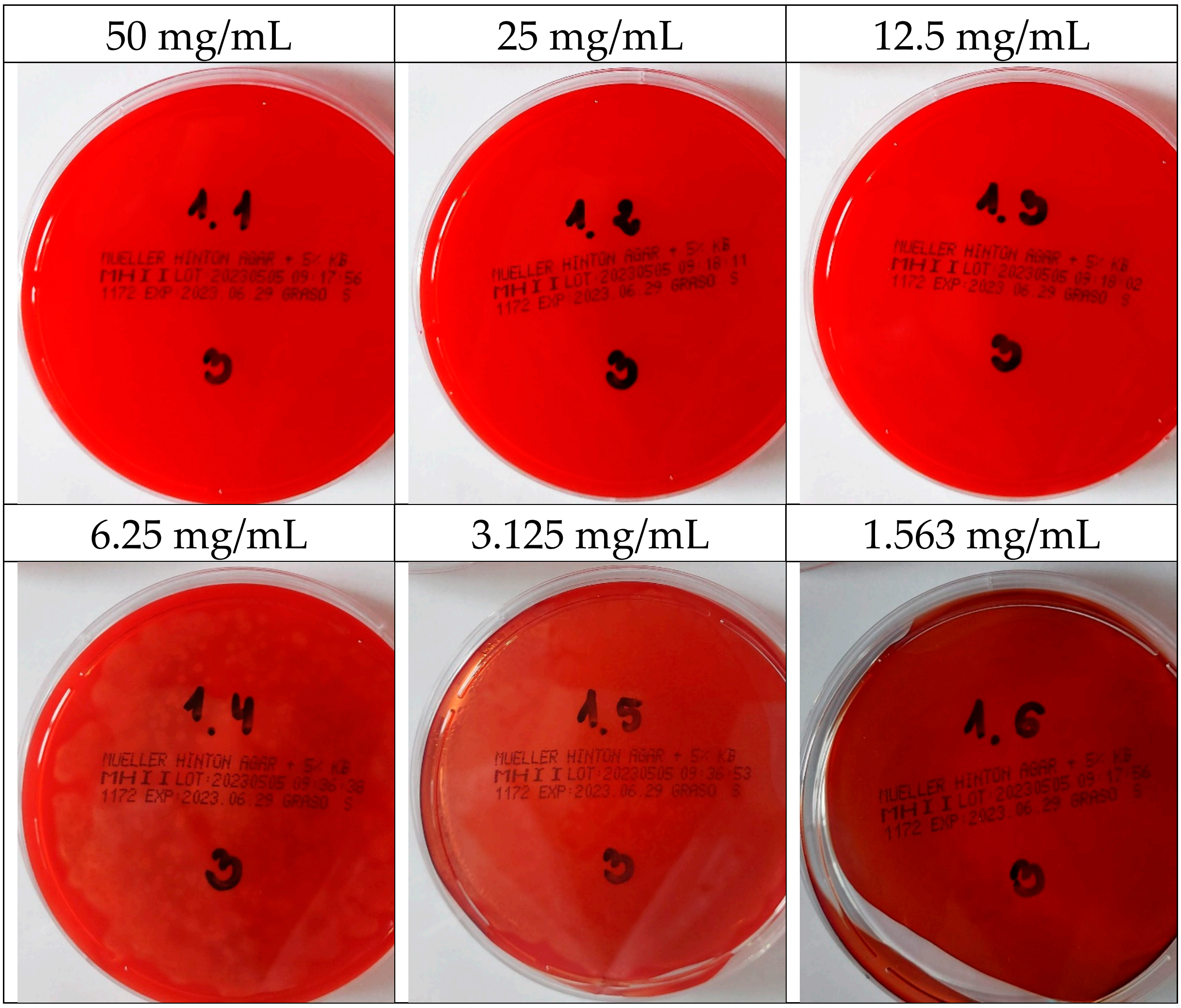

Figure S6. The results of antibacterial activity tests of 20(QA10+TMXDI)$_P$ against *E. coli* (ATCC 25922).

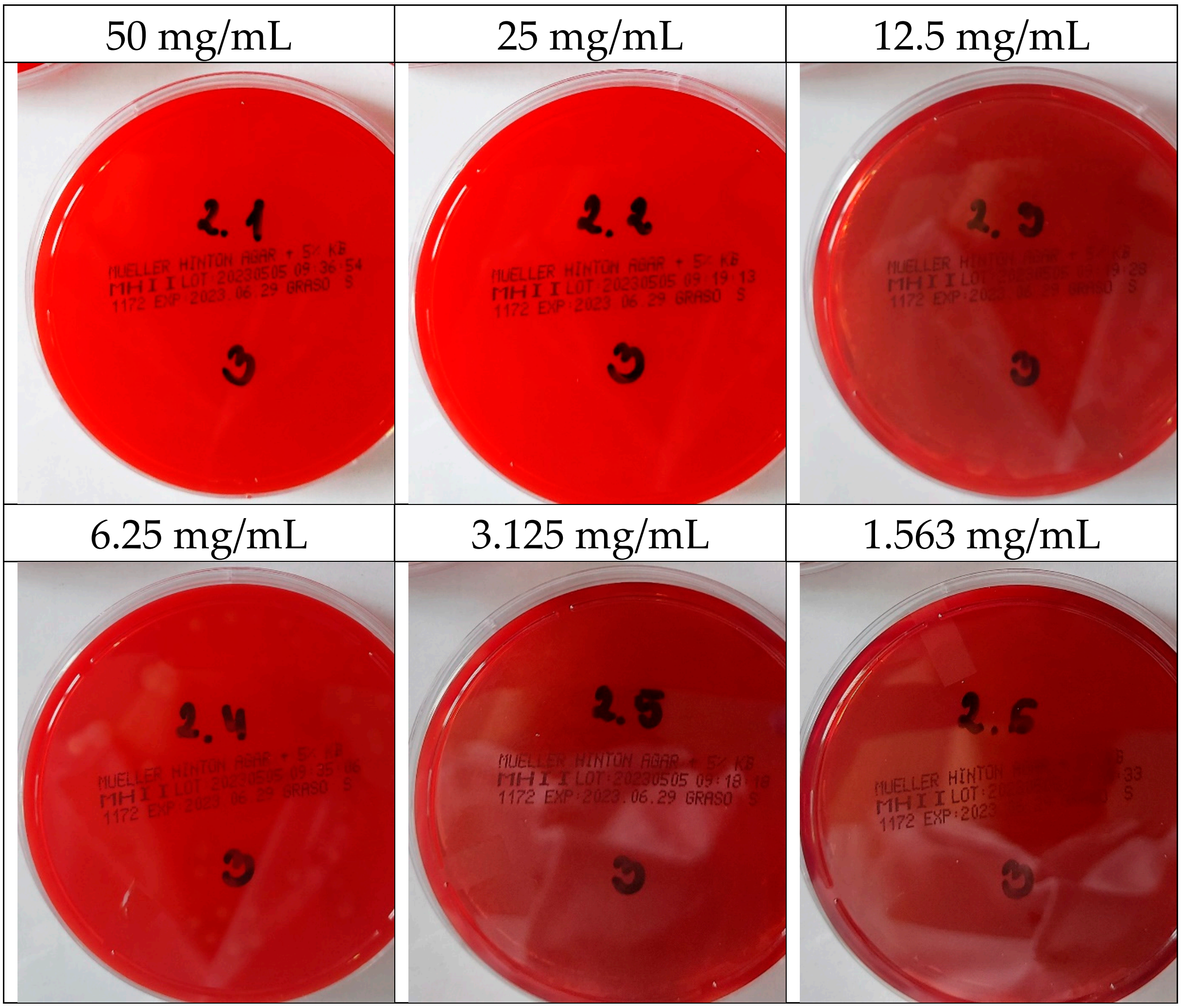

Figure S7. The results of antibacterial activity tests of 20(QA12+TMXDI)$_P$ against *E. coli* (ATCC 25922).

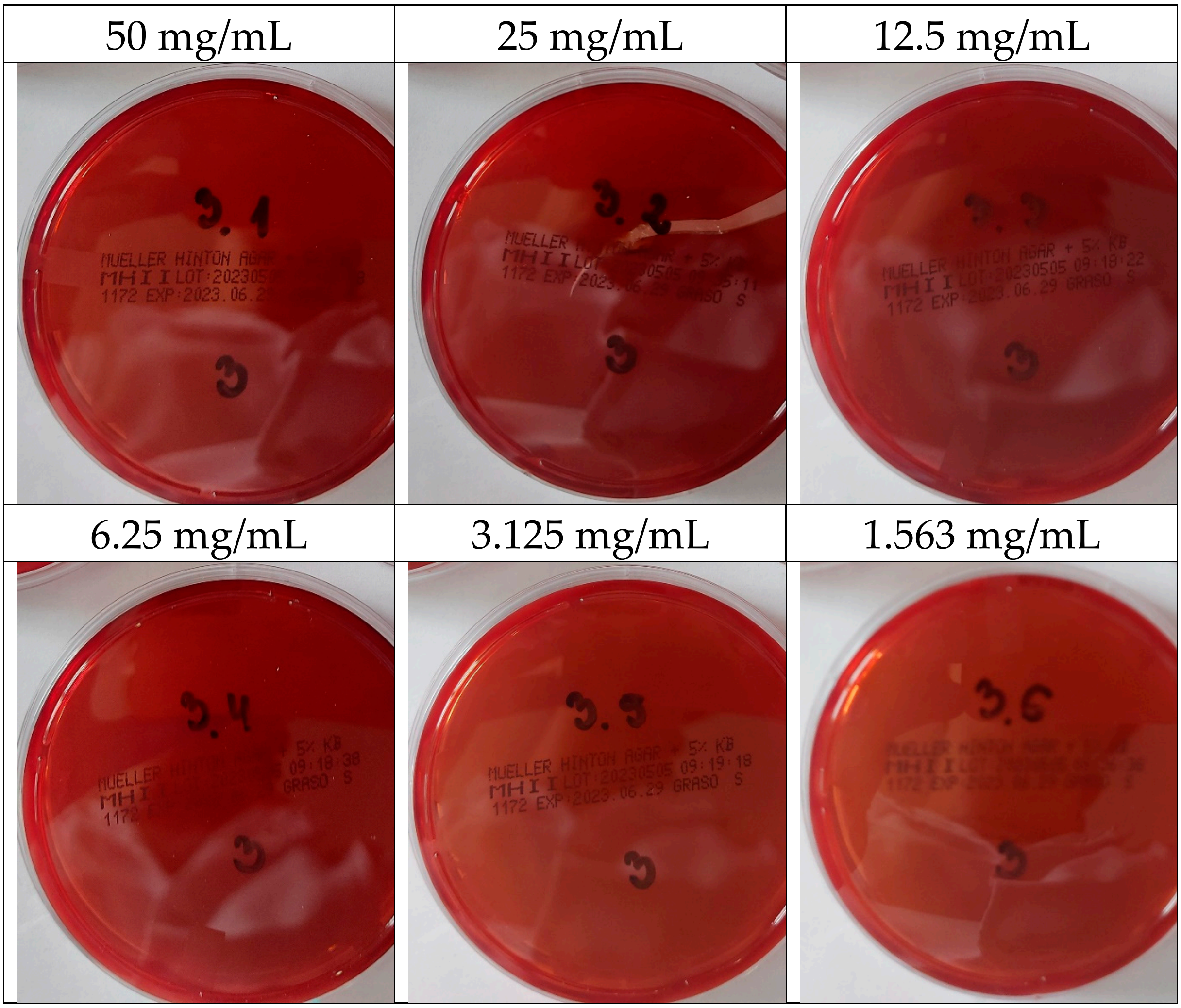

Figure S8. The results of antibacterial activity tests of 40(UDMA)$_P$ against *E. coli* (ATCC 25922).
